# Model-informed dose optimization of carvedilol and nebivolol in cirrhotic patients: a pilot randomized clinical study

**DOI:** 10.1038/s41598-026-56578-3

**Published:** 2026-06-16

**Authors:** Mai Tarek, Ahmed A Ali, Reda Biomy, Khaled Abdelkawy, Eman El-Khateeb

**Affiliations:** 1 Clinical Pharmacy Department, Faculty of Pharmacy, Kafrelsheikh University, Kafrelsheikh, Egypt; 2 Cardiology Department, Faculty of Medicine, Kafrelsheikh University, Kafrelsheikh, Egypt; 3 Simcyp Division, Certara Predictive Technologies (CPT), Sheffield, UK; 4 Clinical Pharmacy Department, Faculty of Pharmacy, Tanta University, Tanta, Egypt

**Keywords:** Antihypertensive drugs, Physiologically based pharmacokinetic (PBPK)-guided dosing, Cirrhosis, Portal hypertension, Cardiology, Diseases, Gastroenterology, Medical research

## Abstract

Liver cirrhosis causes physiological and pharmacokinetic alterations that complicate antihypertensive therapy, in the presence of portal and arterial hypertension. This study evaluated the efficacy and safety of PBPK-guided dosing of carvedilol and nebivolol in cirrhotic patients. First, PBPK models were validated using clinical pharmacokinetic data from healthy volunteers and optimized for cirrhosis, then applied to simulate untested cirrhotic populations and estimate unbound plasma exposure across disease stages. Second, a prospective, open-label, parallel randomized pilot study enrolled 44 cirrhotic patients (Child-Pugh; CP-A or B) with arterial hypertension, who received PBPK-guided doses of carvedilol or nebivolol, with 3 months of follow-up including monthly monitoring of adverse events, blood pressure, heart rate, portal hemodynamics by Doppler ultrasound, and laboratory safety parameters. The model predicted dose reductions of carvedilol 25 mg once daily and nebivolol 10 mg once daily to 11.26 mg and 4.98 mg in mild cirrhosis, 5.52 mg and 2.98 mg in moderate cirrhosis, and 1.99 mg and 1.23 mg in severe cirrhosis. Following administration of doses as close as possible to the PBPK-guided doses in CP A and B, both agents were well tolerated, effectively reducing blood pressure and heart rate without significant changes in hepatic and renal parameters. Portal hemodynamics and platelet count improved in both groups, with carvedilol showing greater effects; adverse events were mild and more frequent with carvedilol. While both drugs controlled blood pressure, carvedilol improved portal hemodynamics more. PBPK-guided dosing addressed pharmacokinetic changes, but pharmacodynamic differences between CP-A and CP-B persisted due to disease progression.

**Supplementary Information:**

The online version contains supplementary material available at 10.1038/s41598-026-56578-3.

## Introduction

Liver Cirrhosis (LC) is the result of chronic inflammation of liver followed by liver fibrosis and formation of nodules due to normal hepatic tissue replacement by fibrotic tissue^1^. One of important complications of LC is portal hypertension (PH) which is defined as a sustained increase in pressure within the portal venous system, which delivers blood from the GI tract and spleen to the liver^2^. Most cases of PH result from chronic liver disorders, in which decreased hepatic sinusoidal blood flow leads to a progressive increase in intrahepatic vascular resistance^3^. This increased pressure also causes enhanced blood flow from the mesenteric circulation, which drains the stomach, intestines, and spleen. Portal hypertension contributes to several cirrhosis-related complications, including ascites and esophageal varices, which can lead to variceal bleeding and hepatic encephalopathy^4^.

Arterial hypertension is a common comorbidity in the general population, and its prevalence among patients with LC varies across different regions^5,6^. Although cirrhosis is generally associated with hemodynamic changes that tend to lower arterial blood pressure, hypertension remains prevalent, particularly in patients with compensated cirrhosis^5–7^. The prevalence rate of arterial hypertension in Egyptian cirrhotic patients is 30.7%^5^. In Egypt, the high prevalence of hepatitis C virus infection, the leading cause of cirrhosis, may contribute to this observation and it is associated with vascular and metabolic disturbances and may induce non‑alcoholic fatty liver disease, leading to systemic inflammation and increased cardiovascular risk^5,8^. Therefore, arterial hypertension remains a clinically relevant condition in Egyptian cirrhotic patients and often necessitates careful blood pressure monitoring and the appropriate use of antihypertensive therapy. However, improper dosing may increase the likelihood of adverse reactions, as these drugs undergo extensive hepatic metabolism and can accumulate in individuals with impaired liver function.

Carvedilol is a non-selective β blocker (NSBB) with additional α_1_-blocking properties and it is primarily used for chronic management of heart failure with reduced ejection fraction, hypertension, and left ventricular dysfunction following myocardial infarction in clinically stable patients^9^. Carvedilol provides both cardiac β blockade and peripheral vasodilation, offering comprehensive cardiovascular benefits^9^. The mainstay of pharmacologic treatment for PH is non-selective β blockers. By lowering cardiac output (β1 blockage) and causing splanchnic vasoconstriction (β2 blockade), which lowers portal venous input, they lower portal pressure^10^. Carvedilol has additional α1-adrenergic blocking effect causes intrahepatic vasodilation and a more significant drop in portal pressure^11^. Meta-analysis evaluating β blockers used in the treatment of PH, including propranolol, carvedilol, and nebivolol, have consistently demonstrated that carvedilol produces the greatest reduction in the Hepatic venous pressure gradient (HVPG) compared with the other agents^12^.

Nebivolol is a third-generation cardio-selective β_1_- adrenergic blocker with nitric oxide (NO)–mediated vasodilatory properties and it is primarily used to treat hypertension and selected cardiovascular conditions because of its high β_1_ -selectivity improves tolerability in patients with pulmonary disease or endothelial dysfunction, while NO-mediated vasodilation helps in vascular conditions like diabetes and erectile dysfunction^13,14^. In patients with LC, endothelial dysfunction leads to impaired NO synthase activity and reduced intrahepatic NO production, which contributes to increased intrahepatic vascular resistance through a dynamic hemodynamic component^15,16^. Consequently, strategies aimed at increasing NO levels within the hepatic circulation may help reduce portal pressure. In a randomized study conducted by Silkauskaitė et al. 2013, nebivolol significantly reduced the HVPG. This effect was sustained after 14 days of treatment. These findings suggest that nebivolol exerts a measurable NO-dependent hemodynamic benefit in PH^15^.

Doppler ultrasonography is a noninvasive method that indirectly evaluates PH by assessing variables such portal vein diameter (PVD), portal vein velocity (PVV), hepatic artery resistance index (HARI), and the existence of portosystemic collaterals. For screening and long-term monitoring of individuals with chronic liver disease, this approach is widely available, safe, and beneficial^17,18^. Despite its specificity and sensitivity, it may vary and can be diminished in patients who have acute hepatic inflammation, ascites, or obesity. Doppler ultrasonography is typically used to identify indirect indicators of PH rather than to give conclusive hemodynamic data because it is unable to accurately measure portal pressure. Despite these drawbacks, it is still a useful instrument for preliminary evaluation, monitoring the course of the illness, and assessing treatment response^19^.

Physiologically based pharmacokinetic (PBPK) modeling and simulation is increasingly used in drug development to support regulatory decisions when clinical data are limited^20,21^. It can also predict pharmacokinetic changes in disease populations, including liver cirrhosis^22^. This approach integrates physiological parameters with drug-specific properties to simulate drug disposition under altered disease conditions and can support dose optimization in both healthy subjects and patients with hepatic impairment^23^. PBPK models can incorporate disease severity using the Child–Pugh classification (A: mild, B: moderate, C: severe)^24^. Among available platforms, Simcyp^®^ is widely used in academia, industry, and regulatory settings for PBPK modeling and simulation^25^.

Accordingly, PBPK modeling provides a rational framework for dose optimization in cirrhotic patients. The present study primarily aimed to evaluate the clinical performance of PBPK-guided carvedilol and nebivolol dosing in cirrhotic patients by assessing their efficacy and safety within each CP- class. This was followed by an inter-treatment comparison between carvedilol and nebivolol to evaluate their relative pharmacodynamic effects and overall clinical performance under PBPK-informed dosing conditions. Finally, an intra-drug comparison between CP- A and CP- B patients was performed within each treatment arm to further characterize the impact of hepatic impairment on pharmacodynamic responsiveness. This approach bridges the gap between computational dose prediction and real-world clinical application, providing preliminary evidence on the feasibility of PBPK-informed dosing strategies in this population.

##  Methods and Patients

This study is divided into two distinct parts: the first one includes validation and simulation of clinical studies using PBPK modelling approach (Simcyp^®^) to predict drug doses of carvedilol and nebivolol in cirrhotic patients, other part includes application of recommended doses of carvedilol and nebivolol in cirrhotic patients.

### Validation and simulation of clinical studies using physiologically pharmacokinetics modelling approach (Simcyp^®^) to predict drug doses of carvedilol and nebivolol in cirrhotic patients

#### Model structure

The PBPK model of carvedilol is modified from Rasool et al. by adding intrinsic solubility 0.02 mg/ml at pH 7.4^26,27^. The PBPK model of nebivolol is obtained from the library of Simcyp^®^ simulator (version 22, Certara, Sheffield, UK). The final list of specific parameters for carvedilol and nebivolol is presented in **Supplementary Tables S1 and S2**.

In summary, developed carvedilol and nebivolol models utilized full PBPK distribution models. The absorption of carvedilol and nebivolol was described by the advanced dissolution, absorption, and metabolism model (ADAM)^28^. For nebivolol, Vss was also predicted following the Rodgers et al.’s method^29,30^. While carvedilol, Vss was predicted by the Poulin and Theil method with the Bierezhkovskiy correction^31^. Carvedilol is mainly bound to albumin and α_1_-acid glycoprotein, while nebivolol is predominantly albumin-bound^32,33^. Carvedilol is metabolized by both phase I and phase II enzymes; phase I metabolism is mediated primarily by CYP P450 isoenzymes CYP2D6, CYP2C9, CYP1A2, and CYP2E1, while phase II metabolism involves conjugation reactions catalyzed uridine 5′‑diphosphate (UDP)‑glucuronosyltransferases, mainly UGT1A1, UGT2B4, and UGT2B7^34,35^. The main metabolic pathways for nebivolol are CYP2D6 and CYP3A4 enzymes^36^.

### Modelling framework

Simulations were conducted for each antihypertensive drug in a healthy volunteer population, and the predicted pharmacokinetics data were compared with observed clinical data. After confirming the model’s ability to accurately predict drug exposure in healthy subjects, it was subsequently extrapolated to estimate exposure in cirrhotic populations classified as CP-A, CP-B, and CP-C. For the cirrhosis populations, specific physiological alterations known to influence drug pharmacokinetics were incorporated into the model as shown in **Supplementary Table S3**.

All simulations for carvedilol validation were conducted according to the study designs reported in the corresponding clinical studies, as summarized in **Supplementary Table S4.** While simulations for validation of nebivolol were performed in previously published study^37^.

The same number of subjects and 10 trials were applied for each simulated clinical study. In cases where no clinical data were available for cirrhotic populations, simulations were performed using 10 subjects per trial, with age ranges of 18–65 years for healthy volunteers and 20–90 years for cirrhotic patients (CP-A, CP-B, and CP-C), with a female proportion of 50%.

### Pharmacokinetic data sources

Pharmacokinetic data for antihypertensive drugs were extracted using the online Plot Digitizer application (Plot Digitizer, Ankit Rohatgi, 2025, Version 3.1.6). Detailed information regarding the reference studies used for the pharmacokinetics of carvedilol in both healthy volunteers and cirrhotic patients is summarized in **Supplementary Table S4.** In carvedilol study, cirrhotic patients were classified on the basis of the cholinesterase level (less than 1800 U/L); 6 patients were classified as CP- C^38,39^. For carvedilol and paroxetine drug interaction study, predicted-to-observed ratios of AUC and Cmax were calculated to assess potential drug–drug interactions (DDIs) using the following Eqs. (1–3).

1 $$\:Predicted\:\mathrm{A}\mathrm{U}\mathrm{C}\:\mathrm{o}\mathrm{r}\:\mathrm{C}\mathrm{m}\mathrm{a}\mathrm{x}\:\mathrm{r}\mathrm{a}\mathrm{t}\mathrm{i}\mathrm{o}\mathrm{s}\:=\frac{\mathrm{P}\mathrm{r}\mathrm{e}\mathrm{d}\mathrm{i}\mathrm{c}\mathrm{t}\mathrm{e}\mathrm{d}\:\mathrm{A}\mathrm{U}\mathrm{C}\:\mathrm{o}\mathrm{r}\:\mathrm{C}\mathrm{m}\mathrm{a}\mathrm{x}\:\mathrm{w}\mathrm{i}\mathrm{t}\mathrm{h}\:\:\mathrm{i}\mathrm{n}\mathrm{h}\mathrm{i}\mathrm{b}\mathrm{i}\mathrm{t}\mathrm{o}\mathrm{r}}{\mathrm{P}\mathrm{r}\mathrm{e}\mathrm{d}\mathrm{i}\mathrm{c}\mathrm{t}\mathrm{e}\mathrm{d}\:\mathrm{A}\mathrm{U}\mathrm{C}\:\mathrm{o}\mathrm{r}\:\mathrm{C}\mathrm{m}\mathrm{a}\mathrm{x}\:\mathrm{w}\mathrm{i}\mathrm{t}\mathrm{h}\mathrm{o}\mathrm{u}\mathrm{t}\:\:\mathrm{i}\mathrm{n}\mathrm{h}\mathrm{i}\mathrm{b}\mathrm{i}\mathrm{t}\mathrm{o}\mathrm{r}}$$

2 $$\:Observed\:\mathrm{A}\mathrm{U}\mathrm{C}\:\mathrm{o}\mathrm{r}\:\mathrm{C}\mathrm{m}\mathrm{a}\mathrm{x}\:\mathrm{r}\mathrm{a}\mathrm{t}\mathrm{i}\mathrm{o}\mathrm{s}\:=\frac{\mathrm{O}\mathrm{b}\mathrm{s}\mathrm{e}\mathrm{r}\mathrm{v}\mathrm{e}\mathrm{d}\:\mathrm{A}\mathrm{U}\mathrm{C}\:\mathrm{o}\mathrm{r}\:\mathrm{C}\mathrm{m}\mathrm{a}\mathrm{x}\:\mathrm{w}\mathrm{i}\mathrm{t}\mathrm{h}\:\:\mathrm{i}\mathrm{n}\mathrm{h}\mathrm{i}\mathrm{b}\mathrm{i}\mathrm{t}\mathrm{o}\mathrm{r}}{\mathrm{O}\mathrm{b}\mathrm{s}\mathrm{e}\mathrm{r}\mathrm{v}\mathrm{e}\mathrm{d}\:\mathrm{A}\mathrm{U}\mathrm{C}\:\mathrm{o}\mathrm{r}\:\mathrm{C}\mathrm{m}\mathrm{a}\mathrm{x}\:\mathrm{w}\mathrm{i}\mathrm{t}\mathrm{h}\mathrm{o}\mathrm{u}\mathrm{t}\:\:\mathrm{i}\mathrm{n}\mathrm{h}\mathrm{i}\mathrm{b}\mathrm{i}\mathrm{t}\mathrm{o}\mathrm{r}}$$

From Eqs. (1) and (2)

3 $$\:Predicted/Observed\:\mathrm{A}\mathrm{U}\mathrm{C}\:\mathrm{o}\mathrm{r}\:\mathrm{C}\mathrm{m}\mathrm{a}\mathrm{x}\:\mathrm{r}\mathrm{a}\mathrm{t}\mathrm{i}\mathrm{o}\mathrm{s}\:=\frac{Predicted\:\mathrm{A}\mathrm{U}\mathrm{C}\:\mathrm{o}\mathrm{r}\:\mathrm{C}\mathrm{m}\mathrm{a}\mathrm{x}\:\mathrm{r}\mathrm{a}\mathrm{t}\mathrm{i}\mathrm{o}\mathrm{s}\:}{Observed\:\mathrm{A}\mathrm{U}\mathrm{C}\:\mathrm{o}\mathrm{r}\:\mathrm{C}\mathrm{m}\mathrm{a}\mathrm{x}\:\mathrm{r}\mathrm{a}\mathrm{t}\mathrm{i}\mathrm{o}\mathrm{s}\:}$$

Paroxetine was selected because it is a well-characterized mechanism-based inhibitor of CYP2D6, allowing verification of the fraction metabolized (fm) by CYP2D6 for carvedilol. The Simcyp library paroxetine compound file was used without More information about paroxetine specific parameters was presented in **Supplementary Table S5.**

### Model performance assessment

The model was assessed by visually comparing the predicted and observed plasma concentration–time profiles, when available. The evaluation focused on whether the observed data were within the 5th and 95th percentiles of the model predictions. In addition, the ratios of predicted to observed pharmacokinetic parameters, including AUC and Cmax, when applicable, were calculated. A ratio within twofold of the observed value was considered acceptable^40,41^.

### Model-based dosing recommendations

In case of nebivolol, dose recommendations for cirrhotic patients were derived from Tarek et al. study using PBPK model^37^. Although a pharmacokinetic study of carvedilol in CP- C patients is available, this study did not propose a specific dosing regimen. Therefore, the unbound area under the plasma concentration–time curve (AUC_unbound_) for each antihypertensive drug was compared between cirrhotic patients and healthy volunteers to evaluate the need for dose adjustment. In addition, total AUC values were also evaluated and reported to provide a comprehensive assessment of systemic drug exposure. This comparison was based on the assumption of a similar exposure–response relationship in both populations. It was applied to the changes in drug exposure across cirrhotic classes relative to healthy subjects.

The recommended dose in cirrhotic patients was estimated based on the following Eq. (4):

$$\:Predicted\:dose\:in\:cirrhosis=$$

4 $$\:\frac{AU{C}_{unbound,last}\:in\:Healthy\:\:Volunteers}{AU{C}_{unbound,last}\:in\:Cirrhotic\:Patients}\times\:Dose\:in\:Healthy\:Volunteers$$

Model simulations were used to predict the relative AUC in cirrhotic patients compared to healthy volunteers. For carvedilol, a dose of 25 mg once daily has shown superior blood pressure–lowering efficacy in systemic hypertension; however, its clinical benefits in patients with cirrhosis for the management of portal hypertension are often limited by adverse drug reactions, particularly at higher doses (25 mg/day)^42–44^. Therefore, PBPK modeling using Simcyp^®^ was employed to explore and optimize an appropriate dosing regimen in cirrhotic patients, aiming to balance efficacy and safety.

## Application of recommended doses of (carvedilol & nebivolol)

### Patients

This study included Egyptian cirrhotic patients with arterial hypertension. Patients were recruited from Out-patients Clinics of Gastroenterology, Hepatology, and Infectious Diseases Department at Kafrelsheikh University Hospital between March 2024 and June 2025. Each participant was assessed at baseline and at follow-up visit 3 months after enrollment. The trial ended after completion of recruitment and follow-up of all participants as planned.

This study was conducted in accordance with the principles of the Declaration of Helsinki. The purpose and nature of the study were explained to all participants, and they were informed of their right to withdraw from the study at any time^45^. The study was approved by the Scientific Research Ethics Committee (Institutional Review Board) of Kafrelsheikh University, Egypt (Approval No. KFSIRB200-126, approved on 29 January 2024). Ethical approval was obtained prior to patient recruitment. Furthermore, the study protocol was registered at ClinicalTrials.gov (Identifier: NCT07397481). Written informed consent was obtained from all patients in this study or their relatives if they couldn’t give consent.

### Application and comparison of recommended doses of carvedilol and nebivolol within CP-classes groups

Primary objective: Evaluation the effect of PBPK- guided doses of carvedilol and nebivolol in each class then compare between carvedilol and nebivolol within CP-classes groups.

**Study design**.

A prospective, open label, randomized parallel pilot clinical study was conducted on fifty cirrhotic patients with arterial hypertension.

- **Inclusion criteria**.

Patients were diagnosed with cirrhosis with comorbid arterial hypertension (defined as systolic blood pressure ≥ 130 mmHg and/or diastolic blood pressure ≥ 80 mmHg) and they have PH without variceal bleeding. Age of patients > 18 years.

b. **Exclusion criteria**.

The following criteria were excluded:

- Patients with kidney disorders or dialysis,
- Hypersensitivity to study medications.
- Active cancer history in the last 2 years.
- Taking drugs that interact with antihypertensive drugs in the last two weeks.
- Pregnancy.
- CP-C cirrhosis.
- Hypertensive emergency criterion (SBP > 180/DBP > 110).

During the screening phase, six patients were excluded because they did not meet the inclusion criteria or fulfilled one or more of the exclusion criteria. Accordingly, forty-four eligible patients were enrolled in the study.

The enrolled patients were classified according to the CP- classification into two main categories: twenty-four patients with CP class A and twenty patients with CP class B. Randomization was performed using a computer-generated sequence within each CP stratum, ensuring balanced allocation between treatment groups. Doses administered as the closest commercially available strengths to the Simcyp^®^ predicted doses, according to CP Classification.

**Child–Pugh Class A**.

Patients with CP class A (*n* = 24) were randomly assigned in a 1:1 allocation ratio using a computer-generated randomization sequence into two equal treatment groups:

- **Group A (n = 12)**: received nebivolol at a dose of 5 mg orally (Nevilob 5 mg^®^, Marcyrl Pharmaceutical Industries, Egypt) once daily to mimic the PBPK simulation regimen of 25 mg once daily in healthy volunteers.
- **Group B (n = 12)**: received carvedilol at a dose of 12.5 mg orally (Carvipress 12.5 mg^®^, Global Napi Pharmaceuticals, Egypt) once daily to mimic the PBPK simulation regimen of 25 mg once daily in healthy volunteers.

**Child–Pugh Class B**.

Patients with CP class B (n = 20) were randomly allocated in a 1:1 allocation ratio using a computer-generated randomization sequence into two equal treatment groups:

- **Group C (n = 10)**: received nebivolol at a dose of 2.5 mg orally (Nevilob 2.5 mg^®^, Marcyrl Pharmaceutical Industries, Egypt) once daily to mimic the PBPK simulation regimen of 25 mg once daily.
- **Group D (n = 10)**: received carvedilol at a dose of 6.25 mg orally (Carvipress 6.25 mg^®^, Global Napi Pharmaceuticals, Egypt) once daily to mimic the PBPK simulation regimen of 25 mg once daily in healthy volunteers.

Allocation concealment was ensured using sequentially numbered, sealed, opaque envelopes prepared by an independent investigator not involved in patient recruitment or assessment, and opened only after enrollment and baseline evaluation, into two equal treatment groups.

### Comparison of drug response across CP-A and CP-B

Secondary objective: Evaluation disease -Stage effect on pharmacodynamic responsiveness

**Primary Outcome Measures**:

The primary outcomes are management of arterial hypertension in cirrhotic patients and portal hypertension in cirrhotic patients.

**Outcome Measures**.

Arterial hypertension in cirrhotic patients is assessed by measuring systolic blood pressure (SBP) [mmHg], diastolic blood pressure (DBP) [mmHg], mean arterial blood pressure (MAP) [mmHg] and heart rate (HR) [bpm] as an indicator of arterial hypertension management. Portal hypertension in cirrhotic patients was assessed by measuring portal vein diameter (PVD)[mm], portal vein velocity (PVV) [cm/s] measured by Doppler ultrasound to assess portal hypertension. PVD and PVV are combined to report congestion Index (CI) (cm/[cm²/s]). Hepatic artery resistance index (HARI) is also measured. PVV and HARI are combined to report Modified liver vascular index (MLVI) [cm²/s]. All parameters are measured at baseline and after 3 months.

**Secondary Outcome Measures**:

Secondary outcomes included preservation of liver function, assessed by changes from baseline in aniline and aspartate aminotransferase enzymes (ALT and AST, U/L), total bilirubin (mg/dL), serum albumin (Alb) [g/dL], and international normalized ratio (INR, unitless) to evaluate coagulation status in cirrhotic patients. Preservation of renal function was assessed by changes from baseline in serum creatinine (Scr, mg/dl) and blood urea nitrogen (BUN, mg/dL). Adverse effects were monitored throughout the study and included headache, gastrointestinal disorders, weakness, shortness of breath, and hyperglycemia. All secondary outcomes were measured at baseline and at the end of the 12-week treatment period except for the adverse effects were assessed monthly.

### Study duration and evaluation

The duration of the study was three months. All patients underwent baseline evaluation before initiation of treatment and were re-evaluated at the end of the 3-month treatment period. The effect of each drug was assessed by comparing pre- and post-treatment parameters within each group. Subsequently, comparisons between carvedilol and nebivolol were performed within each CP class. Before randomization, the study objectives were clearly explained to all participants, and written informed consent was obtained from each patient.

**Doppler Ultrasound**.

Using a Philips EPIQ 7 system equipped with a 3.5 MHz convex transducer (Philips Healthcare, Andover, MA, USA). All assessments were performed with patients in the supine position following a 6-hour fasting period, employing both B-mode and doppler ultrasound. Participants were instructed to hold their breath during Doppler evaluation to optimize measurement accuracy.

All measurements were performed by a radiology specialist, with the probe positioned at the porta Hepatis. A standardized measurement protocol was applied to minimize interobserver variability. The PVD was recorded in millimeters, while the portal vein velocity PVV was expressed in centimeters per second (cm/sec).

Hepatic Artery Resistance Index (HARI) was calculated using the following Eq. (5):

5 $$\:\mathrm{H}\mathrm{A}\mathrm{R}\mathrm{I}=\:\frac{Peak\:Systolic\:Velocity-End\:Diastolic\:Velocity\:}{Peak\:Systolic\:Velocity}$$

HARI; Hepatic Artery Resistance Index.

Congestion index (CI), expressed as (cm/[cm/s^2^]) was calculated using the following Eqs. (6–7)

6 $$\:CI=\frac{Cross\:Sectional\:Area\:\left(CSA\right)}{PVV}$$

7 $$\:CSA={\left(\frac{PVD}{2}\right)}^{2}\times\:\pi\:$$

CI; Congestion Index, PVV; Portal Vein Velocity, CSA; Cross Sectional Area, PVD; Portal Vein Diameter.

Modified Liver Vascular Index (MLVI), expressed in (cm/s), was calculated using the following formula:

8 $$\:MLVI=\frac{PVV}{HARI}$$

MLVI; Modified Liver Vascular Index, PVV; Portal Vein Velocity, HARI; Hepatic Artery Resistance Index.

### **Blood pressure measurement**

Measurements were obtained with the participant in the seated position after at least 5 min of rest, with the arm supported at heart level using mercury sphygmomanometer. An appropriately sized cuff was placed on the upper arm. Two consecutive readings were taken at 1–2-minute intervals, and the average of the two readings was recorded as the final blood pressure value. Blood pressure assessments were performed at baseline and after 2, 4, 8, and 12 weeks of treatment.

### Tests for clinical evaluation

Clinical assessment included serial blood pressure measurements and Doppler ultrasonographic evaluation of portal hemodynamic parameters at baseline and after 12 weeks of treatment. In addition, laboratory investigations were performed to assess treatment efficacy and safety, including liver function tests (ALT, AST, Alb, and total bilirubin), renal function markers (Scr and BUN), coagulation profile (INR), fasting blood glucose (FBG), and complete blood count (CBC). patients will be monitored and any reported adverse effects will be recorded.

### **Statistical analysis**

Values were expressed as mean ± standard deviation (SD). Statistical analysis was performed using IBM SPSS Statistics (Version 31.0; IBM Corp., Armonk, NY, USA). First, normality test is assessed by Shapiro–Wilk test. The paired *t*-test was used to assess within-group differences between baseline and post-treatment values for each treatment arm. Comparisons between carvedilol and nebivolol at baseline and post-treatment were performed within each CP class using an unpaired *t*-test. Comparisons were conducted separately within CP- A and CP- B groups. Comparison between CP-A and CP-B classes were performed using an unpaired *t*-test. Adverse effects and categorical data were evaluated by Fisher’s Exact Test and frequency (percentage).

## Results

The results of this study are presented in two parts. The first part demonstrates the validation and simulations of clinical studies using a PBPK modelling approach (Simcyp^®^) to predict appropriate doses of antihypertensive medications in cirrhotic patients. The second part reports the clinical application and comparison of the recommended doses of selected antihypertensive agents in cirrhotic patients, including evaluation of treatment response and safety.

### Validation and simulations results of clinical studies using physiologically based pharmacokinetics modelling (Simcyp^®^) for antihypertensive dose prediction

#### Assessment of Model in Healthy Subjects

Initially, the developed model for carvedilol was assessed for its ability to predict the pharmacokinetics of carvedilol in healthy volunteers before being applied to cirrhotic patients. Visual inspection indicated a good agreement between the predicted and observed data in healthy subjects, as illustrated **in Supplementary Fig. ****S1****.** Furthermore, the predicted-to-observed ratios of AUC and Cmax, where available, remained within twofold, as shown in **Supplementary Table S6**. In case of carvedilol and paroxetine drug interaction study, all ratios were within two folds as shown in in **Supplementary Table S7**. In case of nebivolol, the validation results were reported by Tarek et al^37^..

### Carvedilol Model Performance in Cirrhotic Patients

Model evaluation for carvedilol was performed in CP-C patients (*n* = 6), taking into account cirrhosis-related physiological changes. Visual inspection indicated a good agreement between the predicted and observed plasma concentration–time profiles as shown in **Supplementary Fig.S2**. Furthermore, predicted-to-observed ratios of AUC and Cmax for both IV infusion and oral carvedilol, comparing healthy subjects to cirrhotic patients, were within a twofold range as shown in **Supplementary Table S8**.

### PBPK-Based Prediction of Drug Exposure in Cirrhotic Populations

Simulations with a 25 mg once-daily dose of carvedilol predicted Total AUC_0−last_ of 393.25 ng·h/ml in healthy volunteers, increasing to 661.27 ng·h/mL in CP-A, 1131.52 ng·h/mL in CP-B, and 2365.23 ng·h/mL in CP-C. This corresponds to a 1.68-, 2.85-, and 6-fold increase in AUC in the CP-A, CP-B, and CP-C populations, respectively, relative to healthy volunteers.

Simulations with a 25 mg once-daily dose of carvedilol predicted an unbound AUC_0−last_ of 2.09 ng·h/mL in healthy volunteers, increasing to 4.65 ng·h/mLin CP-A, 9.47 ng·h/mLin CP-B, and 26.31 ng·h/mL in CP-C. This corresponds to a 2.22-, 4.53-, and 12.59-fold increase in AUC in the CP-A, CP-B, and CP-C populations, respectively, relative to healthy volunteers. The recommended doses for cirrhotic patients were predicted to be to 11.26 mg, 5.52 mg, 1.99 mg in CP-A, CP-B and CP-C respectively.

Similarly, for nebivolol (10 mg once daily), the predicted total AUC_0−last_ increased from 74.76 ng·h/mL in healthy volunteers to 112.54 ng·h/mL in CP-A, 156.95 ng·h/mL in CP-B, and 281.88 ng·h/mL in CP-C, corresponding to 1.5-, 2.2-, and 3.77 -fold increases, respectively, relative to healthy volunteers.

Similarly, for nebivolol (10 mg once daily), as reported by Tarek et al. in a published study, the predicted unbound AUC_0−last_ increased from 1.41 ng·h/ml.

in healthy volunteers to 2.84 ng·h/ml in CP-A, 4.74 ng·h/ml in CP-B, and 11.5 ng·h/ml in CP-C, corresponding to 2-, 3.36-, and 8.16-fold increases, respectively. The recommended doses for cirrhotic patients were predicted to be 4.98 mg, 2.98 mg and 1.23 mg in CP-A, CP-B and CP-C respectively.

Unbound AUCs for both carvedilol and nebivolol in healthy populations and cirrhotic patients are presented in boxplots (GraphPad Software. (2024). GraphPad Prism (Version 10.2.3 [403]) [Computer software]. GraphPad Software, LLC, California, USA) in **Supplementary Fig. S3.**

#### **Application and comparison of recommended doses of** carvedilol and nebivolol

Basal clinical characteristics of study are presented in Supplementary table S9.

##### Application and comparison of recommended doses of carvedilol and nebivolol within CP-classes groups

**Fig. 1:**
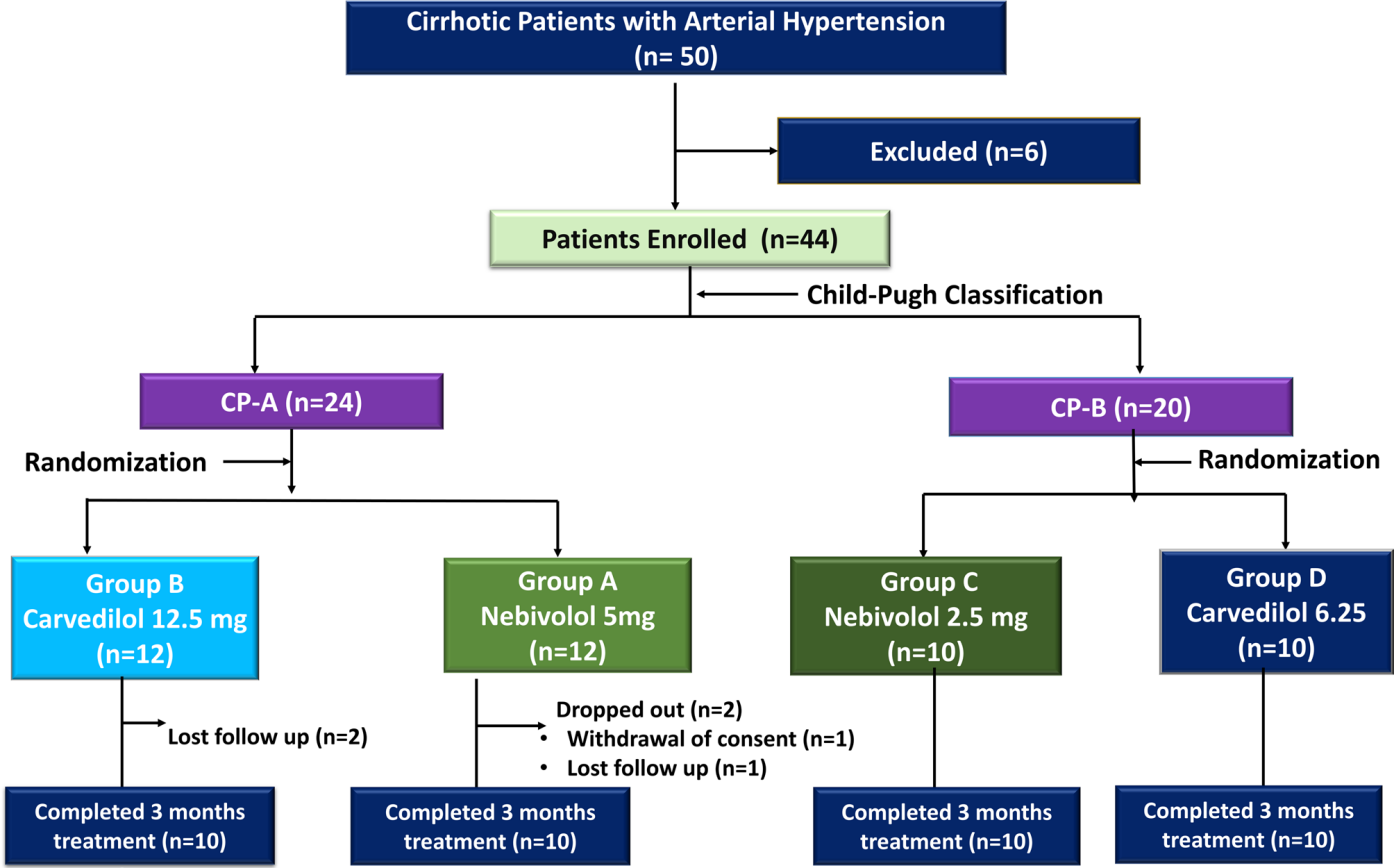
Patient enrollment diagram. CP-A; Child-Pugh A, CP-B; Child PughB.

A total of 44 patients were enrolled and stratified according to CP- class: 24 in class A and 20 in class B. The flow diagram **(**Fig. 1**)** shows the numbers of participants randomized, receiving the intended treatment, and included in the final analysis, along with withdrawals and reasons for losses. During the 3-month study period, four patients dropped out from the CP class A category. In **Group A**, two patients were withdrawn: one due to loss of follow-up and one due to withdrawal of consent. In **Group B**, two patients were lost to follow-up and excluded from the final analysis. All post-treatment values represent measurements obtained at the end of the 3-month treatment period.

The results of the study were statistically analyzed and summarized in the following tables and figures:

**Table 1:** **Demographic characteristics of patients receiving carvedilol and nebivolol in CP- class A and B groups.**

Demographic Data	CP-A (*n* = 20)				CP-B (*n* = 20)			
	Nebivolol (*n* = 10)	Carvedilol (*n* = 10)		Unpaired *P* value	Nebivolol (*n* = 10)	Carvedilol (*n* = 10)		Unpaired *P* value
Age (year)	52.05 ± 5.05	53.8 ± 6.27		0.538	58.6 ± 5.62	59 ± 5.93		0.879
Weight (kg)	74.1 ± 4.1	73.3 ± 4.12		0.670	73.1 ± 4.25	74.8 ± 4.32		0.387
Height (m)	1.74 ± 0.06	1.72 ± 0.06		0.644	1.72 ± 0.06	1.71 ± 0.07		0.918
BMI (kg/m²)	24.57 ± 0.95	24.67 ± 0.9		0.809	25.37 ± 1.17	24.86 ± 1.09		0.331
Gender								
Male (n%)	6(60%)	7 (70%)	1		6(60%)	5 (50%)	1	
Female (n%)	4(40%)	3 (30%)			4(40%)	5 (50%)		
Cause								
Hepatitis C	5 (50%)	6 (60%)	1		7 (70%)	6 (60%)	1	
NAFLD	5 (50%)	4 (40%)			3 (30%)	4 (40%)		

BMI; Body Mass Index. NAFLD; Non-Alcoholic Fatty Liver Disease, CP-A; Child-Pugh A, CP-B; Child-Pugh B. Data represented as **mean ± Standard deviation (unpaired t-test)** or as **Frequency (Percentage) (Fisher’s Exact Test)**.

Within each CP class, there were no significant differences in demographic characteristics between patients receiving carvedilol and those receiving nebivolol. For both Class A and Class B groups, age, weight, height, and BMI were comparable between the two treatment arms (all *P* > 0.05) as shown in Table 1.

In both CP classes, the distribution of gender and underlying causes of LC was similar between the carvedilol and nebivolol groups. In CP- A, males represented 60% in the nebivolol group and 70% in the carvedilol group, while females accounted for 40% and 30%, respectively. In CP- B, the male proportion was 60% for nebivolol and 50% for carvedilol. Regarding etiology, hepatitis C was the most common cause in both classes with no notable differences between treatment groups as shown in Table 1.

**Table 2:** **Comparison of the effects of** carvedilol and nebivolol **on liver**,** renal**,** metabolic**,** hemodynamic**,** and portal hypertension parameters in CP- A and B cirrhotic patients**.

Parameter		CP-A			CP-B		
		Nebivolol 5 mg (*n* = 10)	Carvedilol 12.5mg (*n* = 10)	Unpaired P Value	Nebivolol 2.5 mg (*n* = 10)	Carvedilol 6.25 mg (*n* = 10)	Unpaired P Value
Alb (g/dl)	Baseline	3.56 ± 0.20	3.61 ± 0.18	0.597	3.08 ± 0.12	3.12 ± 0.17	0.552
	The end of 3 months of treatment	3.53 ± 0.18	3.59 ± 0.23	0.496	3.05 ± 0.14	3.11 ± 0.16	0.407
	Paired p-values	0.639	0.837		0.626	0.808	
Bilirubin (mg/dL)	Baseline	1.22 ± 0.18	1.24 ± 0.2	0.864	2.32 ± 0.18	2.31 ± 0.18	0.903
	The end of 3 months of treatment	1.26 ± 0.28	1.25 ± 0.18	0.888	2.28 ± 0.17	2.29 ± 0.15	0.838
	Paired p-values	0.893	0.893		0.663	0.840	
INR	Baseline	1.22 ± 0.11	1.25 ± 0.15	0.620	1.35 ± 0.12	1.36 ± 0.12	0.926
	The end of 3 months of treatment	1.18 ± 0.16	1.23 ± 0.13	0.501	1.32 ± 0.1	1.31 ± 0.2	0.869
	Paired p-values	0.623	0.767		0.442	0.368	
ALT (U/L)	Baseline	40 ± 5.18	38.6 ± 5.04	0.547	30.9 ± 5.38	29 ± 3.89	0.378
	The end of 3 months of treatment	39.75 ± 7.09	37.8 ± 6.11	0.518	30.60 ± 3.53	28.60 ± 4.58	0.288
	Paired p-values	0.836	0.423		0.774	0.775	
AST (U/L)	Baseline	45.1 ± 6.82	43.8 ± 4.66	0.625	39.7 ± 6.6	39.9 ± 8.48	0.954
	The end of 3 months of treatment	45.4 ± 8.94	43.3 ± 6.25	0.550	39.4 ± 6.06	39.55 ± 8.15	0.963
	Paired p-values	0.836	0.644		0.738	0.643	
Scr (mg/dL)	Baseline	0.96 ± 0.15	0.88 ± 0.12	0.209	1.02 ± 0.13	0.94 ± 0.15	0.247
	The end of 3 months of treatment	0.94 ± 0.14	0.93 ± 0.13	0.750	0.99 ± 0.16	0.93 ± 0.17	0.465
	Paired p-values	0.839	0.471		0.584	0.864	
BUN (mg/dL)	Baseline	23.1 ± 3.35	21.4 ± 3.34	0.271	20.8 ± 2.35	21.70 ± 2.16	0.384
	The end of 3 months of treatment	23.25 ± 4.43	21.6 ± 3.98	0.428	21.10 ± 2.23	21.90 ± 3.07	0.514
	Paired p-values	0.898	0.962		0.730	0.811	
FBG (mg/dL)	Baseline	110.95 ± 11.28	109.75 ± 9.58	0.801	102.50 ± 5.24	103.90 ± 4.04	0.512
	The end of 3 months of treatment	111.40 ± 9.94	110.70 ± 9.51	0.874	103.40 ± 5.44	104.30 ± 4.60	0.694
	Paired p-values	0.727	0.383		0.767	0.480	
SBP (mmHg)	Baseline	150.4 ± 5.36	152.1 ± 5.68	0.500	141.9 ± 3.28	142.5 ± 4.72	0.745
	The end of 3 months of treatment	134.3 ± 5.00	135.3 ± 4.37	0.639	128.2 ± 3.62	128.6 ± 4.00	0.817
	Paired p-values	< 0.001*	< 0.001*		< 0.001*	< 0.001*	
DBP (mmHg)	Baseline	88.5 ± 2.59	89.2 ± 3.36	0.608	86.80 ± 3.23	87.7 ± 2.45	0.491
	The end of 3 months of treatment	78.4 ± 2.32	79.2 ± 2.35	0.453	77.00 ± 3.43	78.6 ± 2.72	0.263
	Paired p-values	< 0.001*	< 0.001*		< 0.001*	< 0.001*	
MAP (mmHg)	Baseline	109.13 ± 2.99	110.17 ± 3.88	0.513	105.17 ± 2.98	105.97 ± 3.03	0.559
	The end of 3 months of treatment	97.03 ± 2.26	97.9 ± 2.71	0.448	94.07 ± 2.73	95.27 ± 2.98	0.360
	Paired p-values	< 0.001*	< 0.001*		< 0.001*	< 0.001*	
HR (beats/min)	Baseline	87.2 ± 2.25	87.8 ± 2.20	0.554	90.1 ± 3.54	90.3 ± 3.77	0.904
	The end of 3 months of treatment	76.6 ± 2.55	77.4 ± 2.17	0.459	81.2 ± 2.9	81.00 ± 2.5	0.871
	Paired p-values	< 0.001*	< 0.001*		< 0.001*	< 0.001*	
PVD (mm)	Baseline	13.15 ± 0.39	13.17 ± 0.40	0.933	15.23 ± 0.45	15.26 ± 0.58	0.899
	The end of 3 months of treatment	12.8 ± 0.42	11.9 ± 0.41	< 0.001*	14.95 ± 0.34	14.06 ± 0.59	< 0.001*
	Paired p-values	< 0.001*	< 0.001*		< 0.001*	< 0.001*	
PVV (cm/s)	Baseline	16.75 ± 0.36	16.91 ± 0.60	0.465	14.76 ± 0.45	14.86 ± 0.64	0.689
	The end of 3 months of treatment	17.62 ± 0.36	18.25 ± 0.40	0.002*	15.64 ± 0.37	16.12 ± 0.59	0.042*
	Paired p-values	< 0.001*	< 0.001*		< 0.001*	< 0.001*	
CI (cm²/[cm/s])	Baseline	0.081 ± 0.006	0.081 ± 0.007	0.856	0.124 ± 0.006	0.124 ± 0.013	0.936
	The end of 3 months of treatment	0.073 ± 0.006	0.061 ± 0.005	< 0.001*	0.113 ± 0.004	0.097 ± 0.01	< 0.001*
	Paired p-values	< 0.001*	< 0.001*		< 0.001*	< 0.001*	
HARI	Baseline	0.704 ± 0.024	0.685 ± 0.034	0.721	0.789 ± 0.184	0.779 ± 0.193	0.237
	The end of 3 months of treatment	0.685 ± 0.025	0.656 ± 0.03	0.029*	0.772 ± 0.022	0.745 ± 0.025	0.016*
	Paired p-values	< 0.001*	< 0.001*		< 0.001*	< 0.001*	
MLVI (cm/s)	Baseline	23.83 ± 1.13	24.27 ± 1.79	0.518	18.71 ± 0.33	19.10 ± 1.09	0.288
	The end of 3 months of treatment	25.77 ± 1.20	27.89 ± 1.65	0.004*	20.25 ± 0.43	21.69 ± 1.30	0.004*
	Paired p-values	< 0.001*	< 0.001*		< 0.001*	< 0.001*	
Platelets count (×10³/µL)	Baseline	143.6 ± 5.97	146.3 ± 6.17	0.333	135.8 ± 6.32	139.55 ± 7.21	0.232
	The end of 3 months of treatment	145.1 ± 5.67	151.3 ± 6.64	0.04*	137.16 ± 5.87	143.76 ± 7.96	0.045*
	Paired p-values	< 0.001*	< 0.001*		< 0.001*	< 0.001*	
Hb (g/dL)	Baseline	12.37 ± 0.41	12.22 ± 0.42	0.431	11.23 ± 0.39	11.06 ± 0.37	0.348
	The end of 3 months of treatment	12.41 ± 0.53	12.26 ± 0.61	0.575	11.24 ± 0.49	11.08 ± 0.50	0.480
	Paired p-values	0.680	0.689		0.915	0.889	
WBCs (×10³/µL)	Baseline	5.56 ± 0.46	5.64 ± 0.40	0.667	4.87 ± 0.22	4.69 ± 0.38	0.230
	The end of 3 months of treatment	5.62 ± 0.52	5.74 ± 0.36	0.588	4.89 ± 0.22	4.72 ± 0.28	0.148
	Paired p-values	0.325	0.434		0.675	0.686	

Alb; Albumin INR; International Normalized Ratio, ALT; Alanine Aminotransferase, AST; Aspartate Aminotransferase, Scr; Serum Creatinine, BUN; Blood Urea Nitrogen, FBG; Fasting Blood Glucose, SBP; Systolic Blood Pressure, DBP; Diastolic Blood Pressure, MAP; Mean Arterial Pressure, HR; Heart Rate, PVD; Portal Vein Diameter, PVV; Portal Vein Velocity, CI; Congestion Index, HARI; Hepatic Artery Resistive Index, MLVI; Modified Liver Vascular Index, Hb; Hemoglobin WBCs; White Blood Cells, CP-A; Child-Pugh A, CP-B; Child-Pugh B.* Significant. Data presented as **mean ± Standard deviation**.

Baseline liver function parameters (Alb, bilirubin, INR, ALT, and AST) were comparable between carvedilol and nebivolol groups within each CP- class (*P* > 0.05 for all). Post-treatment values also showed no significant differences between the two drugs, and paired comparisons within each group indicated no significant changes from baseline to after treatment (paired P value > 0.05) as shown in Table 2.

Serum creatinine, BUN, and FBG levels were similar between carvedilol and nebivolol groups at baseline and after treatment in both CP- classes (*P* > 0.05). The changes from baseline were not significantly different between the two drugs, and paired analysis showed no significant within-group changes (paired P value > 0.05) as shown in Table 2.

**Fig. 2:**
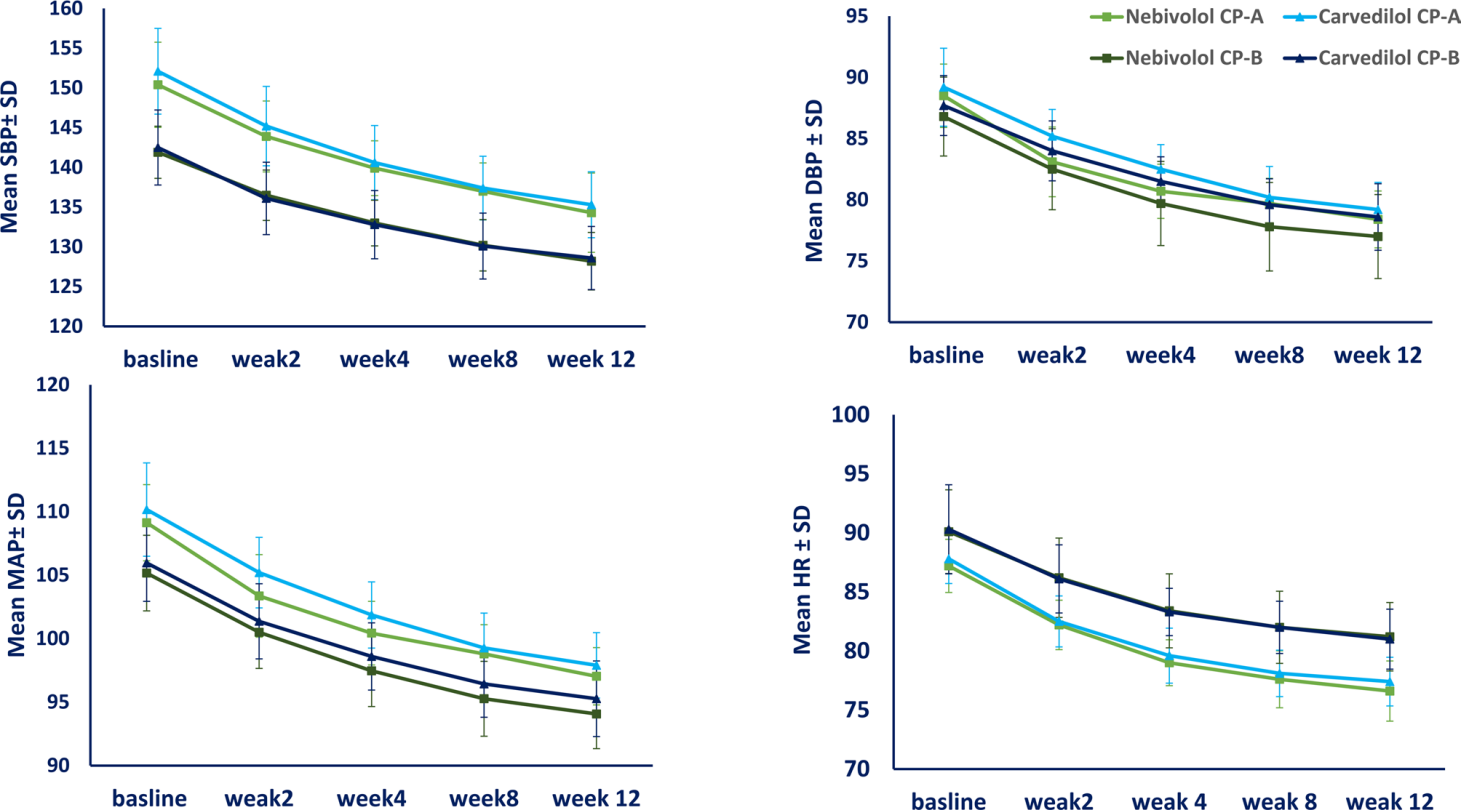
Hemodynamic effects of carvedilol and nebivolol for 12 weeks follow-up period in CP-A and CP-B.

Baseline SBP, DBP, MAP, and HR were comparable between carvedilol and nebivolol groups within each CP- class (all *P* > 0.05). After treatment, no significant differences were observed between the two drugs for any parameter (*P* > 0.05). However, paired analysis within each group showed a highly significant reduction in SBP, DBP, MAP, and HR after treatment compared to baseline (paired P value < 0.001), indicating that both drugs were equally effective in lowering blood pressure and HR. Blood pressure and HR measurements can be presented during 3 months follow up periods in Fig. 2 for CP-A and CP-B.

SBP; Systolic Blood Pressure, DBP; Diastolic Blood Pressure, MAP; Mean Arterial Pressure, HR; Heart Rate, CP-A; Child-Pugh A, CP-B; Child-Pugh B. Data presented as mean ± Standard deviation.

**Fig. 3:**
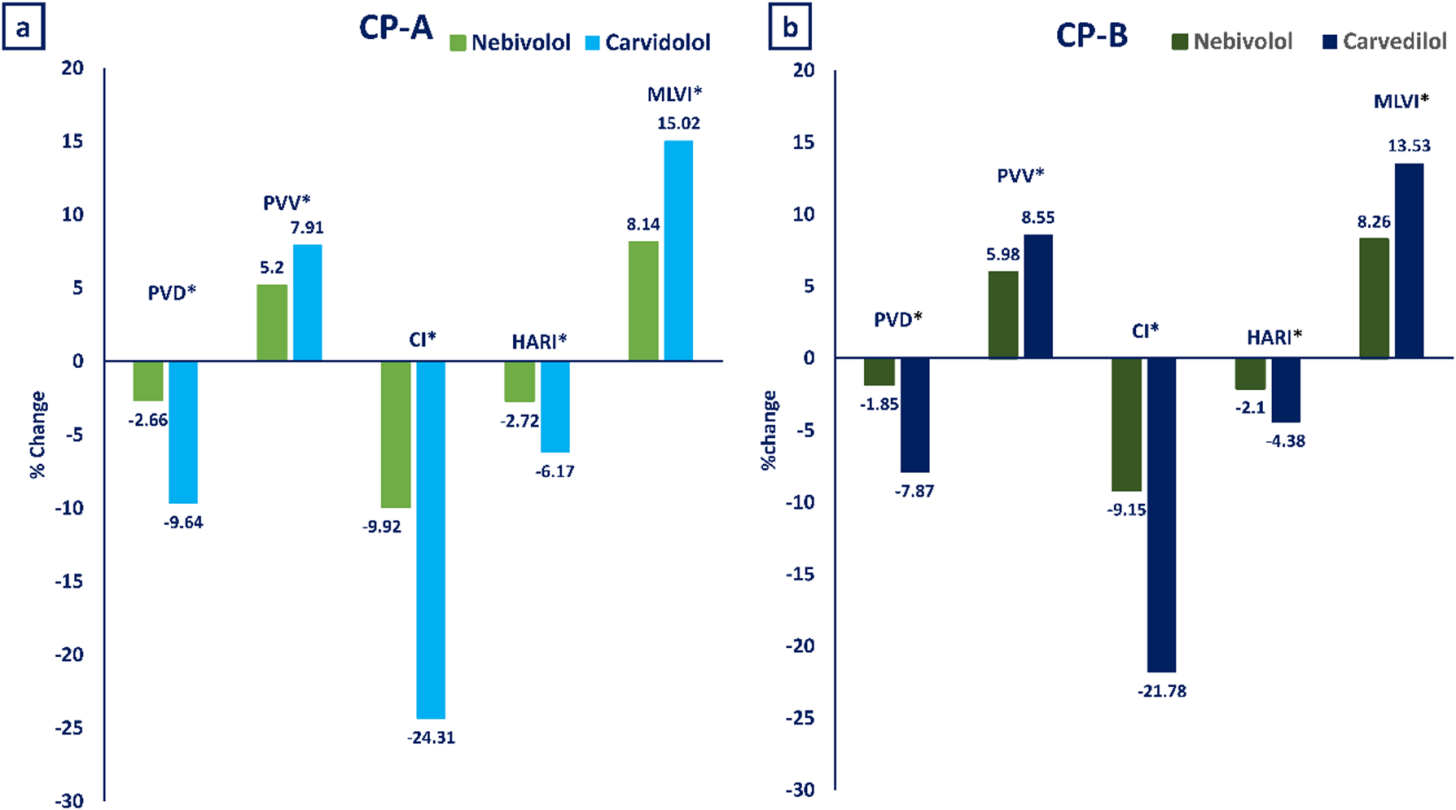
Percentage change in portal hemodynamic parameters with nebivolol vs. carvedilol at the end of the 3-month treatment period: (a) CP-A and (b) CP-B.

Doppler ultrasound assessment revealed that baseline portal hemodynamic parameters (PVD, PVV, CI, HARI, and MLVI) were similar between carvedilol and nebivolol groups within each CP class (all *P* > 0.05). After treatment, significant differences were observed between the two drugs for all parameters (*P* < 0.05), with Carvedilol producing greater reductions in PVD, CI, and HARI and greater increases in PVV and MLVI compared to nebivolol. Paired analysis within each group showed highly significant changes from baseline for all parameters (paired *P* < 0.001). The magnitude of percentage change was significantly larger in the carvedilol group than in the nebivolol group), suggesting a stronger effect of Carvedilol on portal hemodynamics as shown in Fig. 3.

CP-A; Child Pugh A, CP-B; Child Pugh B, PVD; portal vein diameter, PVV; portal vein velocity, CI; congestion index, HARI; hepatic artery resistive index, MLVI; modified liver vascular index, *significant.

**Fig. 4:**
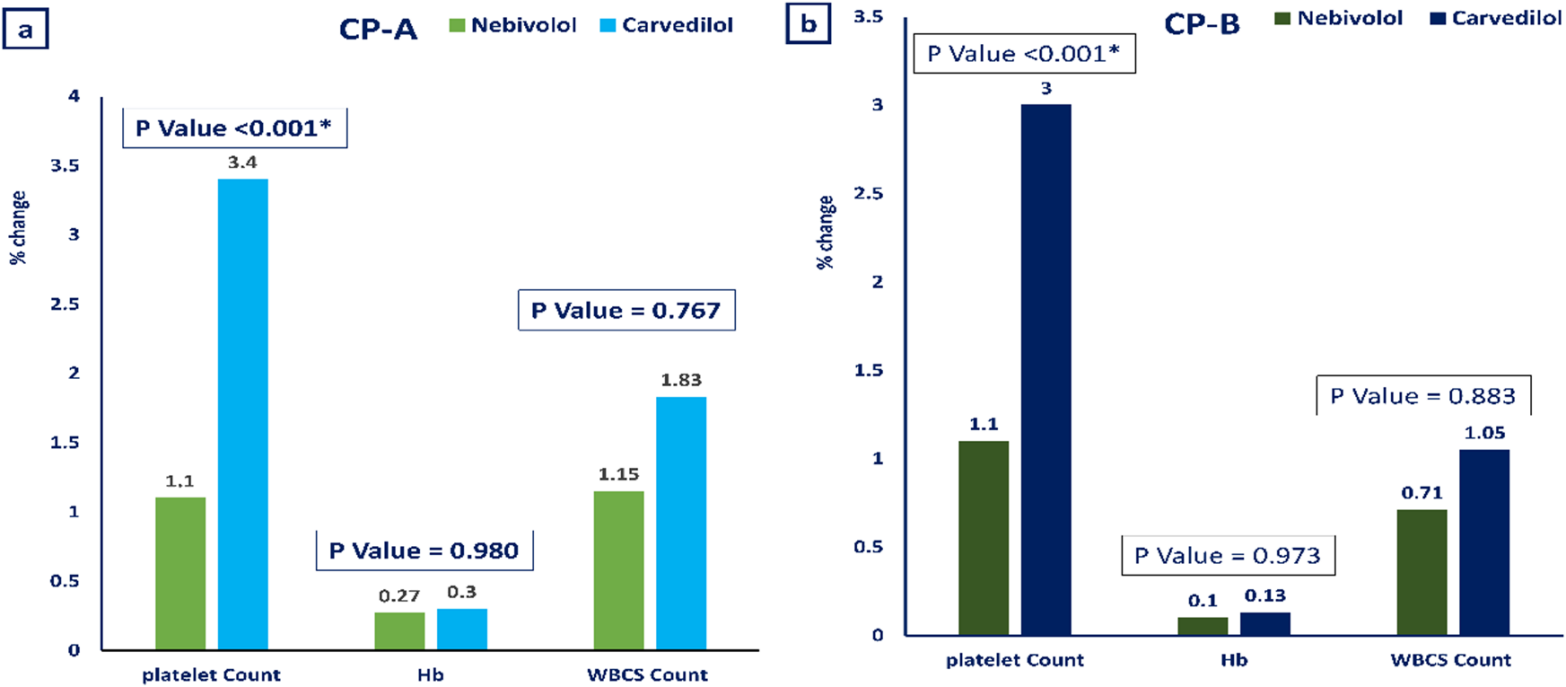
Percentage change in hematological parameters with carvedilol vs. nebivolol at the end of the 3-month treatment period: (a) CP-A and (b) CP-B. CP-A; Child Pugh A, CP-B; Child Pugh B, Hb; hemoglobin, WBCs; white blood cells, *significant.

Baseline hematological parameters (platelet count, Hb, and WBCs) were comparable between carvedilol and nebivolol groups within each CP class (*P* > 0.05). After treatment, platelet count showed a modest increase in both groups, with a greater rise observed in the carvedilol group compared to Nebivolol (*P* < 0.05). Paired analysis confirmed that the increase in platelet count within each group was highly significant (paired *P* < 0.001). In contrast, Hb and WBC counts did not show significant changes after treatment in either group (*P* > 0.05), and between-group differences remained non-significant as shown in Fig. 4.

**Table 3:** Reported adverse effects: carvidolol vs. nebivolol (Child- Pugh A & B).

Adverse effect	CP-A			CP-B		
	Nebivolol 5mg (*n* = 10) *n* (%)	Carvedilol 12.5 mg (*n* = 10) *n* (%)	*P* value	Nebivolol 2.5 mg (*n* = 10) *n* (%)	Carvedilol 6.25 mg (*n* = 10) *n* (%)	*P* value
GIT disorders	**2 (20%)**	**2 (20%)**	**1**	**1 (10%)**	**2 (20%)**	**1**
Headache	**2 (20%)**	**3 (30%)**	**1**	**1 (10%)**	**2 (20%)**	**1**
Weakness	**1 (10%)**	**2 (20%)**	**1**	**0 (0%)**	**1 (10%)**	**1**
Shortness of Breath	**0 (0%)**	**2(20%)**	**0.474**	**0 (0%)**	**1 (10%)**	**1**

Data presented as frequency (percentage) (Fisher’s Exact Test), GIT; Gastrointestinal tract. CP-A; Child-Pugh A, CP-B; Child-Pugh B.

Carvedilol and nebivolol showed similar rates of GI disorders in CP-A but GI disorders occurred in 20% of patients on carvedilol (CP-B) versus 10% on nebivolol. However, headaches, weaknesses and shortness of breath were more frequent with carvedilol. For example, headaches occurred in 30% of patients on carvedilol (CP-A) versus 20% on nebivolol, and shortness of breath was reported only with carvedilol (20% in CP-A and 10% in CP-B).

##### Comparison of drug response across CP-A and CP-B

The results of the study were statistically analyzed and summarized in the following tables:

**Table 4:** Comparison of the hemodynamic and portal hemodynamic effects of carvedilol and nebivolol between CP- A and B cirrhotic patients.

Parameter		Nebivolol			Carvedilol		
		CP-A Nebivolol 5 mg (*n* = 10)	CP-B Nebivolol 2.5 mg (*n* = 10)	Unpaired P Value	CP-A Carvedilol 12.5 mg (*n* = 10)	CP-B Carvedilol 6.25 mg (*n* = 10)	Unpaired P Value
SBP (mmHg)	Baseline	150.40 ± 5.36	141.90 ± 3.28	< 0.001^*^	152.10 ± 5.69	142.50 ± 4.72	< 0.001^*^
	The end of 3 months of treatment	134.30 ± 4.99	128.20 ± 3.62	0.006^*^	135.30 ± 4.37	128.60 ± 3.98	0.002^*^
	% Change	−10.70 ± 1.50	−9.66 ± 1.09	0.093	−11.02 ± 1.30	−9.75 ± 0.80	0.02 ^*^
DBP (mmHg)	Baseline	88.50 ± 2.59	86.80 ± 3.23	0.210	89.20 ± 3.36	87.70 ± 2.45	0.269
	The end of 3 months of treatment	78.40 ± 2.32	77.00 ± 3.43	0.299	79.20 ± 2.35	78.60 ± 2.72	0.604
	% Change	−11.40 ± 1.51	−11.29 ± 2.00	0.895	−11.18 ± 1.35	−10.37 ± 2.12	0.322
MAP (mmHg)	Baseline	109.13 ± 3.00	105.17 ± 2.98	0.008^*^	110.17 ± 3.88	105.97 ± 3.03	0.015^*^
	The end of 3 months of treatment	97.03 ± 2.26	94.07 ± 2.73	0.016^*^	97.90 ± 2.71	95.27 ± 2.98	0.053
	% Change	−11.07 ± 1.10	−10.55 ± 0.85	0.253	−11.11 ± 1.17	−10.10 ± 1.31	0.085
HR (beats/min)	Baseline	87.2 ± 2.25	90.1 ± 3.54	0.042^*^	87.8 ± 2.20	90.3 ± 3.77	0.087
	The end of 3 months of treatment	76.6 ± 2.55	81.2 ± 2.9	0.001^*^	77.4 ± 2.17	81.00 ± 2.5	0.003^*^
	% Change	−12.16 ± 1.64	−9.86 ± 1.30	0.003^*^	−11.83 ± 1.85	−10.26 ± 1.28	0.040 ^*^
PVD (mm)	Baseline	13.16 ± 0.39	15.23 ± 0.45	< 0.001^*^	13.17 ± 0.40	15.26 ± 0.58	< 0.001^*^
	The end of 3 months of treatment	12.81 ± 0.42	14.95 ± 0.34	< 0.001^*^	11.90 ± 0.41	14.06 ± 0.59	< 0.001^*^
	% Change	−2.66 ± 1.50	−1.85 ± 0.91	0.162	−9.64 ± 1.50	−7.87 ± 1.16	0.008 ^*^
PVV (cm/s)	Baseline	16.75 ± 0.36	14.76 ± 0.45	< 0.001^*^	16.92 ± 0.60	14.86 ± 0.64	< 0.001^*^
	The end of 3 months of treatment	17.62 ± 0.36	15.64 ± 0.37	< 0.001^*^	18.25 ± 0.40	16.13 ± 0.59	< 0.001^*^
	% Change	5.20 ± 1.01	5.98 ± 1.16	0.124	7.91 ± 1.58	8.55 ± 1.57	0.378
CI (cm²/[cm/s])	Baseline	0.081 ± 0.006	0.124 ± 0.006	< 0.001^*^	0.081 ± 0.007	0.124 ± 0.013	< 0.001^*^
	The end of 3 months of treatment	0.073 ± 0.006	0.113 ± 0.004	< 0.001^*^	0.061 ± 0.005	0.097 ± 0.01	< 0.001^*^
	% Change	−9.92 ± 2.66	−9.15 ± 1.56	0.440	−24.32 ± 2.45	−21.78 ± 2.21	0.03 ^*^
HARI	Baseline	0.704 ± 0.025	0.789 ± 0.018	< 0.001^*^	0.699 ± 0.035	0.779 ± 0.019	< 0.001^*^
	The end of 3 months of treatment	0.685 ± 0.025	0.772 ± 0.022	0.001*	0.656 ± 0.03	0.7446 ± 0.025	< 0.001^*^
	% Change	−2.72 ± 0.66	−2.10 ± 0.67	0.054	−6.17 ± 1.27	−4.38 ± 1.33	0.007 ^*^
MLVI (cm/s)	Baseline	23.83 ± 1.13	18.71 ± 0.33	0.001*	24.27 ± 1.79	19.10 ± 1.09	< 0.001*
	The end of 3 months of treatment	25.77 ± 1.20	20.25 ± 0.43	< 0.001^*^	27.89 ± 1.65	21.69 ± 1.30	< 0.001*
	% Change	8.14 ± 1.26	8.27 ± 1.52	0.847	15.02 ± 2.15	13.53 ± 1.91	0.119
Platelets count (×10³/µL)	Baseline	143.60 ± 5.97	135.80 ± 6.32	0.011^*^	146.30 ± 6.17	139.55 ± 7.21	0.037^*^
	The end of 3 months of treatment	145.18 ± 5.67	137.26 ± 5.88	0.007^*^	151.30 ± 6.64	143.76 ± 7.69	0.031^*^
	% Change	1.11 ± 0.26	1.09 ± 0.50	0.918	3.41 ± 0.20	3.01 ± 0.54	0.041 ^*^

SBP; Systolic Blood Pressure, DBP; Diastolic Blood Pressure, MAP; Mean Arterial Pressure, HR; Heart Rate, PVD; Portal Vein Diameter, PVV; Portal Vein Velocity, CI; Congestion Index, HARI; Hepatic Artery Resistive Index, MLVI; Modified Liver Vascular Index. Data presented as **mean ± Standard deviation**, CP-A; Child-Pugh A, CP-B; Child-Pugh B.

Baseline analysis demonstrated significant differences between CP-A and CP-B patients in both treatment arms. In both Nebivolol and Carvedilol groups, SBP and MAP were significantly higher in CP-A compared with CP-B patients (*p* < 0.05), while DBP showed no significant baseline difference (*p* > 0.05). Baseline HR differed significantly only in the nebivolol group (*p* = 0.042), whereas no significant difference was observed in the carvedilol group (*p* = 0.087).

All portal hemodynamic indices (PVD, PVV, CI, HARI, and MLVI) showed highly significant baseline differences between CP-A and CP-B patients in both groups (all *p* < 0.001), reflecting more advanced portal hypertension in CP-B cirrhosis. Platelet counts were also significantly lower in CP-B patients at baseline in both treatment arms (*p* < 0.05)All portal hemodynamic indices (PVD, PVV, CI, HARI, and MLVI) showed highly significant baseline differences between CP-A and CP-B patients in both groups (all *p* < 0.001), reflecting more advanced portal hypertension in CP-B cirrhosis. Platelet counts were also significantly lower in CP-B patients at baseline in both treatment arms (*p* < 0.05).

However, between-group comparisons of percentage change showed that carvedilol produced significantly greater improvements in CP-A compared with CP-B patients for SBP, HR, PVD, CI, HARI, and platelet count (*p* < 0.05). In contrast, nebivolol showed largely comparable percentage changes between CP-A and CP-B patients for most parameters, with a significant difference observed only in HR change (*p* = 0.003).

**Table 5:** Reported adverse effects for nebivolol and carvedilol between CP- A and B.

Adverse effect	Nebivolol		*P* Value	Carvedilol		*P* Value
	CP-A Nebivolol 5 mg (*n* = 10) n (%)	CP-B Nebivolol 2.5 mg (*n* = 10) n (%)		CP-A Carvedilol 12.5 mg (*n* = 10) n (%)	CP-B Carvedilol 6.25 mg (*n* = 10) n (%)	
GIT disorders	**2 (20%)**	**1 (10%)**	**1**	**2 (20%)**	**2 (20%)**	**1**
Headache	**2 (20%)**	**1 (10%)**	**1**	**3 (30%)**	**2 (20%)**	**1**
Weakness	**1 (10%)**	**0 (0%)**	**1**	**2 (20%)**	**1 (10%)**	**1**
Shortness of Breath	**0 (0%)**	**0 (0%)**	**----**	**2(20%)**	**1 (10%)**	**1**

Data presented as frequency (percentage) (Fisher’s Exact Test), GIT; Gastrointestinal tract, CP-A; Child-Pugh A, CP-B; Child-Pugh B.

Gastrointestinal disturbances were mild and infrequent, occurring in 20% of CP-A and 10% of CP-B patients in the nebivolol group, and equally in both CP-A and CP-B patients (20%) in the carvedilol group. Headache and weakness were also mild, with slightly higher incidence in CP-A patients in both treatment arms, but without significant between-group differences. Shortness of breath was reported only in the carvedilol group (20% in CP-A and 10% in CP-B) but without significant between-group differences, while no cases were observed in the nebivolol group.

## Discussion

This study employed a PBPK modeling approach to predict and optimize dosing regimens of carvedilol and nebivolol in cirrhotic patients with arterial hypertension. Our simulations suggest that cirrhosis markedly alters drug exposure, with unbound AUC values markedly higher in CP-A, CP-B, and CP-C patients compared to healthy volunteers. These results underscore the need for dose adjustments to achieve therapeutic efficacy while minimizing the risk of adverse events.

Carvedilol and nebivolol are highly plasma protein-bound drugs, with free fractions ($\:{fu,}_{P}$) of 0.0054 and 0.02 respectively^46,47^. for highly protein-bound drugs such as carvedilol and nebivolol, cirrhosis often increases $\:{fu,}_{P}$, which can initially increase total CL. However, cirrhosis also reduces *CL*_*int*_, usually due to decreased hepatic enzyme activity which reduces the total CL. The opposing effects on CL mean that the changes in the total CL or AUC can be masked by the change in plasma protein binding, while unbound AUC (which better reflects pharmacologically active drug) is less affected by changes in binding and is therefore preferred for dose adjustment decisions.

The carvedilol study in patients with CP class C cirrhosis demonstrated an approximately sixfold increase in AUC compared with healthy subjects, which is consistent with the results predicted by PBPK modeling based on total AUC, and our model further extends these results by providing unbound AUC exposure predictions for patients with mild, moderate, severe stages of cirrhosis^39^. A previous PBPK modeling study by Rasool et al. predicted carvedilol doses in cirrhotic patients^27^. However, when using Simcyp^®^ version 22, the predicted doses were slightly different from those reported by Rasool et al. This discrepancy can be attributed to differences in software versions and the updated population data incorporated in the newer Simcyp^®^ release, as proteomic abundance measurements were limited in older versions (version 14), whereas most quantitative proteomics studies providing accurate protein abundance data have only emerged after 2018. The pathophysiological changes for Simcyp^®^ release version 14 were shown as **Supplementary Table S 10**.

Physiologically based pharmacokinetic simulations were used to derive dose recommendations for each CP- class. The investigated antihypertensive drugs were carvedilol and nebivolol. For nebivolol, Simcyp^®^ predicted doses of approximately 4.98 mg for CP-A, which is closest to the commercially available 5 mg tablet; 2.98 mg for CP-B, with 2.5 mg being the nearest available strength; and 1.23 mg for CP-C. However, although PBPK simulations included CP-C patients, they were not enrolled in the clinical study due to safety concerns and the risk of hemodynamic instability in severe hepatic impairment. For carvedilol, the predicted doses were 11.26 mg for CP-A (closest available strength: 12.5 mg), 5.52 mg for CP-B (nearest available: 6.25 mg), and 1.99 mg for CP-C, which was considered not applicable.

The predicted unbound AUC ratios between the nearest commercially and PBPK-predicted doses available doses ranged from 0.84 to 1.13, indicating minimal changes in systemic exposure following dose rounding. These values remained within the commonly accepted bioequivalence range of 0.80–1.25, suggesting that the selected commercial dose strengths produced pharmacokinetic exposures comparable to those predicted by the PBPK simulation as shown **Supplementary Table S 11.**

This study investigated clinically complex scenario in hepatology: patients with LC concurrently experiencing systemic arterial hypertension and PH. To control blood pressure without affecting portal hemodynamics, Careful selection of drugs is required when treating these two conditions. Consequently, carvedilol and nebivolol were compared through Simcyp^®^ guided dosing to determine suitable exposure and safety in hepatic impairment while evaluating their impact on systemic and portal circulatory parameters. “For practical implementation, the commercially available doses were used: carvedilol 12.5 mg and nebivolol 5 mg in CP-A, carvedilol 6.25 mg and nebivolol 2.5 mg in CP-B, as they were the closest to the simulated predictions.

In non-cirrhotic hypertensive populations, beta-blocker therapy with carvedilol and nebivolol has been associated with clinically meaningful reductions in systolic blood pressure generally in the range of approximately 10–20 mmHg and 8–15 mmHg, respectively, with corresponding reductions in diastolic blood pressure of approximately 10–14 mmHg and 7–13 mmHg, respectively^48–50^. In the present study, PBPK-guided dosing in cirrhotic patients achieved systolic blood pressure reductions of approximately 10–20 mmHg and diastolic blood pressure reductions of approximately 7–12 mmHg, which generally fall within the ranges previously reported in non-cirrhotic populations. This suggests that despite substantial dose reduction in cirrhosis, the observed antihypertensive effect remains consistent with the expected pharmacodynamic response of these agents.

In both CP- A and B patients, carvedilol and nebivolol significantly reduced SBP and DBP, MAP, and HR, without inducing clinically significant changes in hepatic or renal biochemical markers. These findings confirm the cardiovascular efficacy and short-term biochemical safety of both agents in individuals with cirrhosis. Our result is consistent with randomized crossover trial in hypertensive subjects found both agents to be similarly effective in lowering SBP and DBP, confirming comparable hemodynamic efficacy^51^.

Carvedilol demonstrated superior enhancement in doppler ultrasound parameters associated with PH. It resulted in a more significant decrease in PVD and CI, as well as a more pronounced increase in PVV and MLVI. Nebivolol also had beneficial effects, but it is less pronounced compared to that of carvedilol on portal hemodynamics.

The changes in Doppler ultrasound seen in this study show that portal circulation is getting better. A smaller PVD and CI, along with a higher PVV, suggest that there is less congestion in the portal vein and better blood flow through it^52^. An increase in MLVI means that the hepatic vascular state is more balanced, while a decrease in HARI may mean that normal hepatic arterial buffering has returned^52^.

These non-invasive results confirm previous invasive studies indicating that carvedilol results in more significant decreases in the HVPG relative to other β blockers, including nebivolol and propranolol^12^. Meta-analyses of NSBBs used to treat PH, such as propranolol, carvedilol, and nebivolol, have consistently shown that carvedilol produces the greatest reduction in the HVPG compared with the other agents. These findings align with our study results, which indicated that carvedilol is more effective than nebivolol in enhancing portal hemodynamics and Doppler parameters. This consistency strengthens the clinical significance of our comparison and corroborates the efficacy of carvedilol as an effective treatment for lowering portal pressure in cirrhotic patients.

A third-generation selective β1-blocker with nitric oxide-mediated vasodilatory effects, nebivolol has also demonstrated the capacity to reduce portal pressure, albeit not to the same extent as carvedilol. Nebivolol decreased HVPG from 19.7 mmHg (SD, 2.5) at baseline to 15.7 mmHg (SD, 2.6) after 60 min (20.1% reduction) and to 16.7 mmHg (SD, 3.2) after 14 days (15.3% reduction) in the randomized study by Silkauskaitė, V. et al^15^.. These findings support a clinically relevant portal pressure–lowering effect of nebivolol, most likely through an increase in the hepatic circulation Nitric oxide bioavailability. However, the lower nebivolol lresponse rate (28% vs. 88% after 14 days) in comparison to carvedilol indicates that it would be a better option for patients who are intolerant to NSBBs rather than first-line medication. However, research on animals has shown conflicting results, showing that in cirrhotic rat models, higher dosages (10 mg/day) of nebivolol paradoxically raise portal pressure because of increased portal inflow and excessive splanchnic vasodilation (Reiberger et al., 2013)^53^. This difference emphasizes the complexity of NO signaling in cirrhosis and emphasizes the importance of more study to clarify the function of nebivolol and optimize dosage approaches.

Following three months of carvedilol medication, our study showed a substantial decrease in PVD, suggesting better portal hemodynamics and less congestion. These results align with earlier studies. For instance, Ahmed et al. found that carvedilol reduced the diameter of the portal vein from 12.77 ± 0.633 mm at baseline to 10.49 ± 0.953 mm after 24 weeks, demonstrating that it can lower portal pressure by combining mild α_1_-blockade with NSBBs^54^. The observed trend is consistent with these long-term findings, supporting the significance of carvedilol in enhancing doppler parameters and portal circulation even though our follow-up duration was shorter (three months).

There was a modest elevation in platelet count with both medications, but the carvedilol group had a greater rise. People with cirrhosis often have thrombocytopenia because of hypersplenism produced by PH^55^. Consequently, the observed increase in platelet count may reflect reduced splenic sequestration in association with changes in portal hemodynamics^55^. These findings support the potential utility of platelet-based non-invasive indicators in the assessment of PH. Our findings are in line with those reported by Mujahid et al., who observed that patients with hepatitis C-related cirrhosis treated with carvedilol showed a noticeable improvement in platelet count^56^. This may suggest a possible association between carvedilol therapy, modulation of portal hemodynamics, and changes in platelet count; The observed platelet increase may reflect multiple mechanisms including reduced splenic sequestration secondary to lowered portal pressure, and possible changes in thrombopoietin levels, though mechanistic confirmation was not possible because specific biomarkers were not assessed^57^. At baseline, most systemic and portal hemodynamic parameters significantly differed between CP-A and CP-B patients in both the carvedilol and nebivolol groups, reflecting the greater severity of circulatory and portal hemodynamic alterations in CP-B cirrhosis. However, DBP did not significantly differ between CP-A and CP-B patients in either treatment group, and baseline HR showed no significant difference in the carvedilol groups. These findings may be attributed to overlap in systemic hemodynamic status between cirrhosis stages, interindividual variability, compensatory hyperdynamic circulation, and the limited statistical power associated with the pilot study sample size.

For nebivolol, PBPK-guided dose adjustment produced generally comparable responses between CP-A and CP-B patients for most systemic and portal hemodynamic parameters, suggesting successful exposure normalization across cirrhosis severity classes. However, HR reduction remained significantly greater in CP-A patients. This finding may be attributed to the more pronounced autonomic dysfunction and impaired β-adrenergic responsiveness in CP-B cirrhosis, which can attenuate chronotropic responsiveness despite optimized drug exposure.

For carvedilol, although PBPK-guided dose optimization reduced the potential for excessive exposure in CP-B patients, several parameters, including SBP, HR, PVD, CI, HARI, and platelet count, still demonstrated significantly greater improvement in CP-A patients. This may reflect the more advanced portal hypertensive state and structural hepatic alterations in CP-B cirrhosis, which can limit vascular and portal hemodynamic responsiveness to carvedilol therapy. In addition, the combined non-selective β-blocking and α1-blocking properties of carvedilol produce hemodynamic effects that are highly dependent on vascular reactivity and intrahepatic resistance, both of which are more severely impaired in advanced cirrhosis.

In contrast, platelet count changes with Nebivolol were comparable between CP-A and CP-B patients. This may be due to its weaker impact on portal hemodynamics compared with carvedilol, as its selective β1-blockade results in less reduction of intrahepatic resistance and portal pressure, leading to a limited effect on hypersplenism-related thrombocytopenia across both Child–Pugh classes.

Therefore, while PBPK-guided dosing successfully accounted for pharmacokinetic alterations associated with cirrhosis severity, residual differences in pharmacodynamic responsiveness between CP-A and CP-B patients persisted, particularly for parameters closely linked to portal vascular remodeling and circulatory dysfunction. The primary purpose of the PBPK-guided dosing strategy was exposure normalization and preservation of efficacy/safety within each cirrhosis stage rather than achieving identical absolute PD responses across stages.

The absence of clinically significant or treatment-limiting adverse events in both Child–Pugh groups suggests that PBPK-guided dose reduction in CP-B patients may have effectively prevented excessive systemic exposure. Importantly, expected dose-related β-blocker toxicity manifestations, including severe hypotension, symptomatic bradycardia (heart rate < 50 bpm), or clinically relevant intolerance, were not observed.

This finding is particularly relevant in advanced cirrhosis, where impaired hepatic clearance and altered pharmacokinetics may predispose patients to drug accumulation and hemodynamic instability. The comparable safety profile between CP-A and CP-B patients, despite the administration of lower doses in CP-B, provides indirect clinical support for the appropriateness of the PBPK-informed dosing strategy.

Notably, these results contrast with previously reported cases of carvedilol-induced cardiogenic shock in cirrhotic patients, where administration of 25 mg was associated with severe toxicity despite the absence of classical overdose conditions^58^.In the present study, no such severe adverse events were observed, which may be attributed to PBPK-guided dose reduction in CP-B patients (6.25 mg versus 12.5 mg in CP-A), thereby likely preventing excessive systemic exposure and mitigating the risk of clinically significant β-blocker toxicity.

In general, both medications were well tolerated. Headache, weakness and dyspnea were among the most common adverse effects of carvedilol, which are in line with its pharmacological effects^59^. These findings emphasize the necessity of regular blood pressure monitoring and cautious dose titration, particularly in individuals with low baseline MAP.

Limitation.

The sample size was relatively small, which may hinder the generalizability of the results. Further studies with large sample size may be required in the future to confirm our preliminary data. Doppler ultrasound parameters, while non-invasive and practical, serve solely as indirect indicators and cannot completely substitute for direct HVPG measurement. However, it is important to note that our results were in agreement with HVPG results, which show that the Doppler-based method is reliable. Doppler ultrasonography was performed by a single radiology specialist; while this minimizes interobserver variability, the absence of a second blinded reader limits external verification of measurements.

In the clinical phase of this study, the efficacy and safety of both carvedilol and nebivolol were not evaluated in patients with CP class C cirrhosis. Therefore, further studies are warranted to investigate and optimize dosing strategies for both agents in patients with liver cirrhosis, particularly those with advanced (CP-C) disease.

The mechanisms underlying changes in platelet count were not fully explored, as thrombopoietin levels and other related biomarkers were not assessed.

Although the carvedilol PBPK model adequately predicted exposure ratios (AUC and Cmax fold changes) within the accepted 2-fold criteria across populations (including healthy subjects and cirrhotic CP class C), it did not consistently capture absolute pharmacokinetic values, particularly Cmax in healthy volunteers. Therefore, the model is more suitable for predicting relative exposure changes rather than precise absolute concentrations, and further refinement is required to improve absolute Cmax predictions. This discrepancy may be related to the rapid absorption phase in healthy subjects not being fully captured by the ADAM model, plausibly due to underestimation of intestinal permeability or formulation-related absorption effects.

The pharmacokinetic parameters and unbound exposure estimates reported for CP-A and CP-B populations were based on PBPK simulations and were not validated using measured plasma drug concentrations from the enrolled patients. Furthermore, since the study population consisted of outpatient cirrhotic patients, intensive serial pharmacokinetic sampling was not feasible within the scope of the current pilot study. A follow-up study with sparse pharmacokinetic sampling and population PK analysis is planned to clinically validate the PBPK-derived exposures.

## Conclusion

Physiologically based pharmacokinetic guided dose reductions of carvedilol and nebivolol produced clinically meaningful antihypertensive efficacy with an acceptable safety profile in CP-A and B cirrhotic patients, supporting the feasibility of model-informed dosing in this population. As a secondary observation, carvedilol showed greater improvements in portal hemodynamic parameters than nebivolol; however, this finding should be interpreted cautiously given the limited sample size and the study’s design and may reflect the distinct pharmacological properties of carvedilol rather than differences in dosing strategy. The clinical efficacy and safety observed across CP- A and B classes are consistent with successful exposure normalization by PBPK-guided dosing, although pharmacokinetic measurements in patients are needed for direct confirmation. However, residual pharmacodynamic differences between CP-A and CP-B patients persisted, likely reflecting progressive portal vascular remodeling and circulatory dysfunction in advanced cirrhosis.

## Supplementary Information

Below is the link to the electronic supplementary material.

Supplementary Material 1

## Data Availability

The data that support the findings of this study are available from the corresponding author upon reasonable request.

## Declarations

### Accordance Approval

This study was conducted in accordance with the principles of the Declaration of Helsinki. The purpose and nature of the study were explained to all participants, and they were informed of their right to withdraw from the study at any time.

### Registration trial

The study was registered retrospectively at ClinicalTrials.gov (Identifier: NCT07397481) on 4 February 2026.
