# Supplementary material for: Model-informed dose optimization of carvedilol and nebivolol in cirrhotic patients: a pilot randomized clinical study
Source: Sci Rep. 2026 Jun 16;16:18663. doi: 10.1038/s41598-026-56578-3 (PMC13269781; doi:10.1038/s41598-026-56578-3)
Supplement: Supplementary file 1 — Supplementary Material 1 [file 41598_2026_56578_MOESM1_ESM.docx]

**Model-Informed Dose Optimization of Carvedilol and Nebivolol in Cirrhotic Patients: A Pilot Randomized Clinical Study**

**Mai Tarek^1, $^, Ahmed AAli^1^, Reda Biomy^2^, Khaled Abdelkawy^1^, Eman El-Khateeb^3,4^**

**^1^** Clinical Pharmacy Department, Faculty of Pharmacy, Kafrelsheikh University, Kafrelsheikh, Egypt.

^2^ Cardiology Department, Faculty of Medicine, Kafrelsheikh University, Kafrelsheikh, Egypt

^3^ Certara Predictive Technologies (CPT), Simcyp Division, Sheffield, UK

^4^ Clinical Pharmacy Department, Faculty of Pharmacy, Tanta University, Tanta, Egypt.

^$^ Corresponding author

Mai Tarek

Email: [mai.tareq@pharm.Kfs.edu.eg](mailto:mai.tareq@pharm.Kfs.edu.eg)

Supplementary Table S1: Drug-specific parameters for carvedilol

| Parameter | Value/model | Reference |
| --- | --- | --- |
| Physicochemical properties | | |
| Molecular weight | 406.47 | PubChem |
| LogP_o/W_ | 4.19 |  |
| PKa | 7.97 | ^1^ |
| Blood/ plasma B:P ratio | 0.69 | ^2^ |
| Absorption | | |
| Model | ADAM | ^3^ |
| P_eff, man_ ($\boldsymbol{10}^{\boldsymbol{-4}}$cm/s) | 1.94 | ^4^  Predicted after calibrating with metoprolol and propranolol using Simcyp® |
| Permeability assay | Caco-2 |  |
| $\boldsymbol{P}_{\boldsymbol{appaA:B}}$ ($\boldsymbol{10}^{\boldsymbol{-6}}$cm/s) | 13.46 |  |
| Intrinsic Solubility (mg/mL) | 0.02 at pH 7.4 | ^5^ |
| Model | Full PBPK | |
| Vss (L/kg) | 1.69 | Predicted |
| Prediction method | Method1: Paulin and Theil method with the Bierzhkovskiy correction | ^6^ |
| $\boldsymbol{fu,}_{\boldsymbol{P}}$ | 0.0054 | ^2^ |
| Elimination | | |
| CYP2D6 CLᵢₙₜ (µL/min/mg/pmol) | 339.7 | Simcyp® retrograde model of enzyme kinetics |
| CYP1A2 CLᵢₙₜ (µL/min/mg/pmol) | 8.71 |  |
| CYP2C9 CLᵢₙₜ (µL/min/mg/pmol) | 3.1 |  |
| CYP2E1 CLᵢₙₜ (µL/min/mg/pmol) | 3.71 |  |
| UGT1A1 CLᵢₙₜ (µL/min/mg/pmol) | 4.04 | Simcyp® parameter estimation module^7^ |
| UGT2B4 CLᵢₙₜ (µL/min/mg/pmol) | 3.43 |  |
| UGT2B7 CLᵢₙₜ (µL/min/mg/pmol) | 5.75 |  |
| $\boldsymbol{CL}_{\boldsymbol{r}}$ (L/h) | 0.25 | ^8^ |

LogP_o/W_; octanal-water partition coefficient, pKa; acid dissociation constant, gut blood flow, $P_{appaA:B};$ apparent permeability from Apical to Basolateral, Vss; volume of distribution at steady state, B:P; blood to plasma, ${fu,}_{P}$*;*; fraction of unbound drug in plasma P_eff,man_; human jejunum permeability, CYP cytochrome P450, UGT uridine diphosphate glucuronosyl transferase

Supplementary Table S2: Drug-specific parameters for nebivolol

| Parameter | Value/model | Reference |
| --- | --- | --- |
| Physicochemical properties | | |
| Molecular weight | 405.44 | PubChem |
| LogP_o/W_ | 4.18255 |  |
| PKa | 8.9 | Chemaxon |
| Blood/ plasma B:P ratio | 0.626 | Predicted in Simcyp® ® |
| Absorption | | |
| Model | ADAM | ^3^ |
| P_eff,man_ ($\boldsymbol{10}^{\boldsymbol{-4}}$cm/s) | 0.69114 | Predicted |
| Permeability assay | MechPeff model | |
| Distribution | | |
| Model | Full PBPK Model | |
| $\boldsymbol{V}_{\boldsymbol{SS}}$(L/kg) | 10.059 | Predicted by Rodger’s et al |
| Prediction method | Method 2 (Rodger’s et al) | Simcyp® ® file |
| $\boldsymbol{fu,}_{\boldsymbol{P}}$ | 0.02 | ^9^ |
| Elimination | | |
| CYP2D6 $\boldsymbol{CL}_{\boldsymbol{int}}$ (µL /min/pmol) | 579.4 | optimised to match observed AUC after a single 5 mg oral dose in healthy volunteers |
| CYP3A4 $\boldsymbol{CL}_{\boldsymbol{int}}$ (µL /min/pmol) | 0.43 |  |
| Additional Clearance Liver HLM $\boldsymbol{CL}_{\boldsymbol{int}}$ (µL/min/mg protein) | 125.2 |  |

LogP_o/W_; _octonal_-water partition coefficient, pKa; acid dissociation constant, B:P; blood to plasma, Vss; volume of distribution at steady state, ${fu,}_{P}$*;*fraction of unbound drug in plasma P_eff,man_; human jejunum permeability, CL_int_; intrinsic clearance, ADAM; advanced dissolution absorption and metabolism model, MechPeff model; mechanistic permeability model, PBPK; physiologically based pharmacokinetics, CYP; cytochrome P450, HLM; human liver microsome, AUC; area under the curve

Supplementary Table S3: Pathophysiological changes between healthy populations and cirrhotic patients with different classes applied in carvedilol PBPK modelling.

| Parameter | Gender | Population | | | | Reference |
| --- | --- | --- | --- | --- | --- | --- |
|  |  | Healthy | CP-A | CP-B | CP-C | All population specific parameter values were kept the same  As in Simcyp® ® simulator (version 22, Certara, Sheffield, UK). |
| Hepatic arterial blood flow rate (%) | Male / female | 6.5 | 8.41 | 8.19 | 9.37 |  |
| Portal blood flow rate (%) | Male | 19 | 19.69 | 15.85 | 12.71 |  |
|  | Female | 21.5 | 22.28 | 17.93 | 14.38 |  |
| Villous blood flow rate (%) | Male / female | 6 | 8.85 | 9.16 | 11.15 |  |
| Cardiac output scalar | Male/ female | 1 | 1.15 | 1.31 | 1.4 |  |
| Liver volume (L) | Male / female | 1.6506 | 1.4195 | 1.1719 | 0.97383 |  |
| (Alb) (g/L) | Male | 50.34 | 42.61 | 35.97 | 28.52 |  |
|  | Female | 49.38 | 41.8 | 35.28 | 27.97 |  |
| Hematocrit (%) | Male | 43 | 41.1 | 34.75 | 33.92 |  |
|  | Female | 38 | 36.32 | 30.71 | 29.97 |  |
| Mean fasting gastric residence time (h) | Male | 0.4 | 0.4 | 0.56 | 0.6 |  |
| Hepatic enzymes or transporter Abundance | | | | | |  |
| CYP2D6 (pmol/mg/protein) | | 9.4 | 8.43 | 5.88 | 1.59 |  |
| CYP1A2 (pmol/mg/protein) | | 52 | 37.6 | 17.2 | 8.81 |  |
| CYP2C9 (pmol/mg/protein) | | 77.7 | 77.7 | 77.7 | 44.2 |  |
| CYP2E1 (pmol/mg/protein) | | 61 | 32.8 | 39.2 | 32.8 |  |
| UGT1A1 (pmol/mg/protein) | | 43 | 36.97 | 36.97 | 25.99 |  |
| UGT2B4 (pmol/mg/protein) | | 54 | 35.86 | 24.61 | 11.26 |  |
| UGT2B7(pmol/mg/protein) | | 71 | 22.9 | 21.83 | 22.92 |  |

CYP; cytochrome, UGT; Uridine Diphosphate-Glucuronosyltransferase

Supplementary Table S4: Clinical characteristics and design of the studies used for drug model validation of carvedilol.

| Drug | Population | Number of subjects | Age range (years) | Weight (kg)  (range) | Proportion of females | Dose (mg) | Route | Reference |
| --- | --- | --- | --- | --- | --- | --- | --- | --- |
| Carvedilol | **Healthy** | **8** | **53-65** | **75-90** | **0** | **25mg (SD)** | **Oral** | **^10^** |
|  |  |  |  |  |  | **50 mg (SD)** | **Oral** |  |
|  |  |  |  |  |  | **50 mg (SD) with food** | **Oral** |  |
|  |  |  |  |  |  | **5 mg over 5 minutes** | **IV Infusion** |  |
|  | **Healthy (10 Caucasian)** | **10** | **46.7-65*** | **70.8-101*** | **0.2** | **12.5 mg once daily for 4 weeks (MD)** | **Oral** | **^11^** |
|  | **Healthy (9 Caucasian and 2 African American)** | **11** | **38.9-49.7*** | **70.7-93.1*** | **0.273** | **25 mg once daily for 4 weeks (MD)** | **Oral** |  |
|  | **Healthy (12 Caucasian)** | **12** | **35.7-54.9** | **65.6-115.8** | **0.083** | **50 mg once daily for 4 weeks (MD)** | **Oral** |  |
|  | **Healthy** | **13** | **33-65** | **64-112** | **0.307** | **12.5 mg was administered daily for 2 days followed by a 7-day course of**  **25 mg also administered daily** | **Oral** | **^8^** |
|  | **Healthy** | **12** | **20-50** | **ND** | **0.5** | **25 mg (SD)** | **Oral** | **^12^** |
|  | **Healthy** | **20** | **19-45** | **60-92** | **0** | **12.5 mg over one hour.** | **IV Infusion** | **^13^** |
|  | **Healthy** | **18** |  |  |  | **25 mg (SD)** | **Oral** |  |
|  | **Healthy** | **19** |  |  |  | **50 mg (SD)** |  |  |
|  | **Cirrhotic patient (CP-C)** | **6** | **40-76** | **65.5-96** | **0.167** | **12.5 mg over one hour.** | **IV infusion** | **^13^** |
|  |  |  |  |  |  | **25 mg (SD)** | **Oral** |  |
| Carvedilol with paroxetine | **Healthy** | **11** | **18-45** | **ND** | **0.364** | **Paroxetine (10mg) is administrated once daily for two days, then twice daily for five days, then twice daily on the day of carvedilol 12.5 mg dosing then daily for 4 days** | **Oral** | **^14^** |

IV; Intravenous, CP-C; Child‒Pugh, * Ranges used in simulations, SD; Single Dose, MD; Multiple Dose, ND; Not Defined in the study


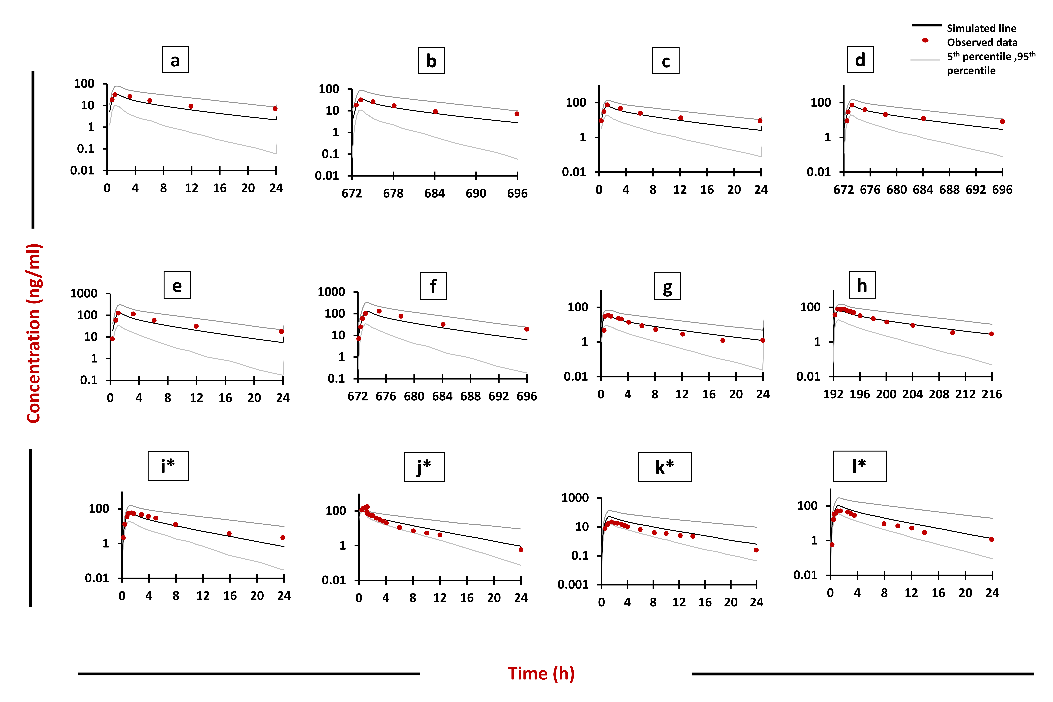


Supplementary Fig.1 Plasma concentration-time profiles of carvedilol after a) Oral dose of 12.5 mg was administrated on day 1(10 trials of 10 male subjects, 46.7-65, 20% females) ^11^. b) Oral dose of 12.5 mg was administrated after 4 weeks (10 trials of 10 male subjects, 46.7-65, 20% females) ^11^. c) Oral dose of 25 mg was administrated on day 1 (10 trials of 11 subjects, 38.9-49.7, 27.3% females)^11^. d) Oral dose of 25 mg was administrated after 4 weeks (10 trials of 11 subjects, 38.9-49.7, 27.3% females) ^11^. e) Oral dose of 50 mg was administrated on day 1 (10 trials of 11 subjects, 38.9-49.7, 27.3% females) ^11^. f) Oral dose of 50 mg was administrated after 4 weeks (10 trials of 12 subjects, 35.7-54.9, 8.3% females) ^11^. g) Oral dose of 12.5 mg was administered on day 1 (10 trials of 13 subjects, 33-65, 30.7% females) ^8^. h) oral dose of 25 mg was administered on day 9 (10 trials of 13 subjects, 33-65, 30.7% females) ^8^. i) A single oral dose of 25 mg (10 trials of 12 subjects, 20- 50 years, 50% females) ^12^. j) A single IV infusion dose of 12.5 mg over one hour (10 trials of 20 male subjects, 19- 45 years) ^15^. k) A single oral dose of 25 mg (10 trials of 18 male subjects, 19- 45 years) ^15^. l) A single oral dose of 50 mg (10 trials of 19 male subjects, 19- 45 years) ^15^. Solid black lines represent the simulated average concentration, red dots represent the observed clinical data, and grey lines represent the 5^th^ percentile and the 95^th^ percentiles. * Concentration presented as median

Supplementary Table S5: Drug-specific parameters for paroxetine

| Parameter | Value/model | Reference |
| --- | --- | --- |
| Physicochemical properties | | |
| Molecular weight | 329.40 | PubChem |
| LogPo/w | 3.55 | ^16^ |
| pKa | 9.66 |  |
| Blood/plasma B:P ratio | 1.26 | ^17^ |
| Absorption | | |
| Model | ADAM | ^3^ |
| P_eff, man_ ($\boldsymbol{10}^{\boldsymbol{-4}}$cm/s) | 183 | Predicted |
| Permeability assay | Mechanistic model | |
| Distribution | | |
| Model | Minimal PBPK | ^17^ |
| Vss (L/kg) | 12.48 |  |
| $\boldsymbol{fu,}_{\boldsymbol{P}}$ | 0.05 |  |
| Elimination | | |
| CYP1A2 Vmax (pmol/min/pmol) | 0.271 | ^17^ |
| CYP1A2 Km (µM) | 8.8 |  |
| CYP2C19 Vmax (pmol/min/pmol) | 0.6 |  |
| CYP2C19 Km (µM) | 26 |  |
| CYP2D6 Vmax (pmol/min/pmol) | 7.275 |  |
| CYP2D6 Km (µM) | 0.028 |  |
| CYP3A4 Vmax (pmol/min/pmol) | 1.272 |  |
| CYP3A4 Km (µM) | 13.3 |  |
| CYP3A5 Vmax (pmol/min/pmol) | 0.384 |  |
| CYP3A5 Km (µM) | 108 |  |
| Additional HLM CLint (µL/min/mg protein) | 61.9 |  |
| CLr (L/h) | 0.5 |  |
| Drug–drug interaction parameters |  |  |
| CYP2D6 Ki (µM) | 0.46 | ^17^ |
| CYP2D6 MBI K_app_ (µM) | 0.066 |  |
| CYP2D6 MBI K_inact_ (1/h) | 10.2 |  |
| CYP3A4 Ki (µM) | 32.7 |  |
| CYP3A4 MBI K_app_ (µM) | 4.03 | ^17,18^ |
| CYP3A4 MBI K_inact_ (1/h) | 0.66 | ^17^ |

LogP_o/W_; _octanol_-water partition coefficient, pKa; acid dissociation constant, B:P; blood to plasma, Vss; volume of distribution at steady state, ${fu,}_{P}$*;*fraction of unbound drug in plasma P_eff,man_; human jejunum permeability, CL_int_; intrinsic clearance, ADAM; advanced dissolution absorption and metabolism model, MechPeff model; mechanistic permeability model, PBPK; physiologically based pharmacokinetics, CYP; cytochrome P450, HLM; human liver microsom , Vmax; maximum velocity; Km; Michaelis–Menten constant; CLr; renal clearance; Ki; inhibition constant; MBI; mechanism-based inhibition; Kapp; apparent inactivation constant; Kinact; maximum inactivation rate constant.

**Supplementary Table S6: Predicted to observed pharmacokinetics parameters of carvedilol**

| Drug | PK parameters | Predicted | Observed | P/O | Observed data Reference |
| --- | --- | --- | --- | --- | --- |
| Carvedilol (Oral )25 mg (SD) | AUC_0-48_ (ng·h/mLmL) | 393.88 | 337 | 1.17 | ^10^ |
|  | Cmax (ng·mL) | 66.96 | 67 | 0.999 |  |
| Carvedilol (Oral )50 mg (SD) | AUC_0-48_ (ng·h/mL) | 776.11 | 717 | 1.1 |  |
|  | Cmax (ng·mL) | 128 | 122 | 1.05 |  |
| Carvedilol (Oral )50 mg (SD) with food | AUC_0-48_ (ng·h/mL) | 991.42 | 741 | 1.34 |  |
|  | Cmax (ng·mL) | 144.23 | 128 | 1.13 |  |
| Carvedilol (IV Infusion) 5 mg over 5 minutes | AUC_0-48_ (ng·h/mL) | 237 | 184 | 1.34 |  |
| Carvedilol (Oral) 12.5 mg (First Dose) (10 Caucasian) | AUC_0-∞_ (ng·h/mL) | 244.04 | 180 | 1.36 | ^11^ |
|  | Cmax (ng/mL) | 34.79 | 39 | 0.89 |  |
| Carvedilol (Oral) 12.5 mg (Last Dose) (10 Caucasian) | AUC_0-24_(ng·h/mL) | 247.85 | 184 | 1.35 |  |
|  | Cmax (ng/mL) | 37.58 | 32 | 1.17 |  |
| Carvedilol (Oral) 25 mg (First Dose) (9 Caucasian and 2 African American) | AUC_0-∞_ (ng·h/mL) | 384.09 | 410 | 0.94 |  |
|  | Cmax (ng/mL) | 65.55 | 75 | 0.874 |  |
| Carvedilol (Oral) 25 mg (Last Dose) (9 Caucasian and 2 African American) | AUC_0-24_ (ng·h/mL) | 385.59 | 343 | 1.12 |  |
|  | Cmax (ng/mL) | 68.20 | 73 | 0.93 |  |
| Carvedilol (Oral )50 mg (First Dose) (12 Caucasian) | AUC_0-∞_ (ng·h/mL) | 793.82 | 1097 | 0.72 |  |
|  | Cmax (ng/mL) | 130.94 | 161 | 0.81 |  |
| Carvedilol 50 (Oral) mg (Last Dose) (12 Caucasian) | AUC_0-24_ (ng·h/mL) | 800.53 | 1136 | 0.7 |  |
|  | Cmax (ng/mL) | 136.74 | 167 | 0.82 |  |
| Carvedilol (Oral) 12.5 mg (day1) | AUC_0-24_ (ng·h/mL) | 181.76 | 165 | 1.1 | ^8^ |
|  | Cmax (ng/mL) | 31.36 | 46.7 | 0.67 |  |
| Carvedilol (Oral) 25 mg (day 9) | AUC_0-24_ (ng·h/mL) | 389.78 | 413 | 0.94 |  |
|  | Cmax (ng/mL) | 65.20 | 104 | 0.63 |  |
| Carvedilol (Oral) 25 mg (SD) | AUC_0-24_ (ng·h/mL) | 260.50^*^ | 272^*^ | 0.96 | ^12^ |
|  | Cmax (ng/mL) | 58.07^*^ | 72^*^ | 0.81 |  |
| Carvedilol (Oral) 50 mg (SD) | AUC_0-24_ (ng·h/mL) | 486.26^*^ | 348^*^ | 1.4 | ^15^ |
|  | Cmax (ng/mL) | 103.63^*^ | 66^*^ | 1.57 |  |

AUC_0-∞_; area under the concentration–time curve from time zero to infinity, AUC_0-t_ ; area under the concentration–time curve from zero to specific time, Cmax; maximum plasma concentration, SD; single dose, MD; multiple doses, IV; intravenous P/O; predicted over observed ratio, ^*^ median.

| Drug | Interacting drug | Predicted without interaction | | Predicted with interaction | | Observed without interaction | | Observed with interaction | | Predicted with/without interaction | | Observed with/without interaction | | Predicted/observed | | Observed  Reference |
| --- | --- | --- | --- | --- | --- | --- | --- | --- | --- | --- | --- | --- | --- | --- | --- | --- |
|  |  | **AUC** | **Cmax** | **AUC** | **Cmax** | **AUC** | **Cmax** | **AUC** | **Cmax** | **AUC ratio** | **Cmax ratio** | **AUC**  **ratio** | **Cmax ratio** | **AUC**  **ratio** | **Cmax**  **ratio** |  |
| Carvedilol | **Paroxetine** | 197.08 | 33.07 | 661.62 | 72.08 | 80.1 | 26.3 | 185.8 | 46.7 | 3.36 | 2.2 | 2.32 | 1.8 | **1.45** | **1.22** | **^14^** |

**Supplementary Table S7: Model verification of the carvedilol–paroxetine drug–drug interaction (DDI), presenting predicted-to-observed ratios for key pharmacokinetic parameters.**

AUC; area under the curve(ng·h/mL), Cmax; maximum concentration (ng/mL)

Supplementary Table S8: Predictive performance of carvedilol model in healthy and cirrhotic patients.

| Drug | Predicted (healthy) | | Predicted (cirrhotic patients) | | Observed (healthy volunteers) | | Observed (cirrhotic patients) | | Predicted ratio (cirrhotic to healthy) | | Observed ratio  (cirrhotic to healthy) | | Predicted/observed  Disease impact | | Reference |
| --- | --- | --- | --- | --- | --- | --- | --- | --- | --- | --- | --- | --- | --- | --- | --- |
|  | **AUC** | **Cmax** | **AUC** | **Cmax** | **AUC** | **Cmax** | **AUC** | **Cmax** | **AUC ratio** | **Cmax ratio** | **AUC**  **Ratio** | **Cmax ratio** | **AUC ratio** | **Cmax**  **ratio** |  |
| Carvedilol (IV infusion) | 420.57* | 158.21* | 876* | 139.14* | 354* | 173* | 531.28* | 150* | 2.1 | 1.13 | 1.5 | 0.87 | **1.4** | **1.3** | **^13^** |
| Carvedilol  (Oral) | 243* | 51.07** | 1471.61* | 130.31* | 157** | 21* | 884.75* | 104* | 6.1 | 2.6 | 5.6 | 4.9 | **1.1** | **1.9** |  |

AUC; area under the curve(ng·h/mL), Cmax; maximum concentration (ng/mL), IV; intravenous * median ** Predicted Cmax in healthy volunteers after oral administration exceeded the 2-fold acceptance range

**
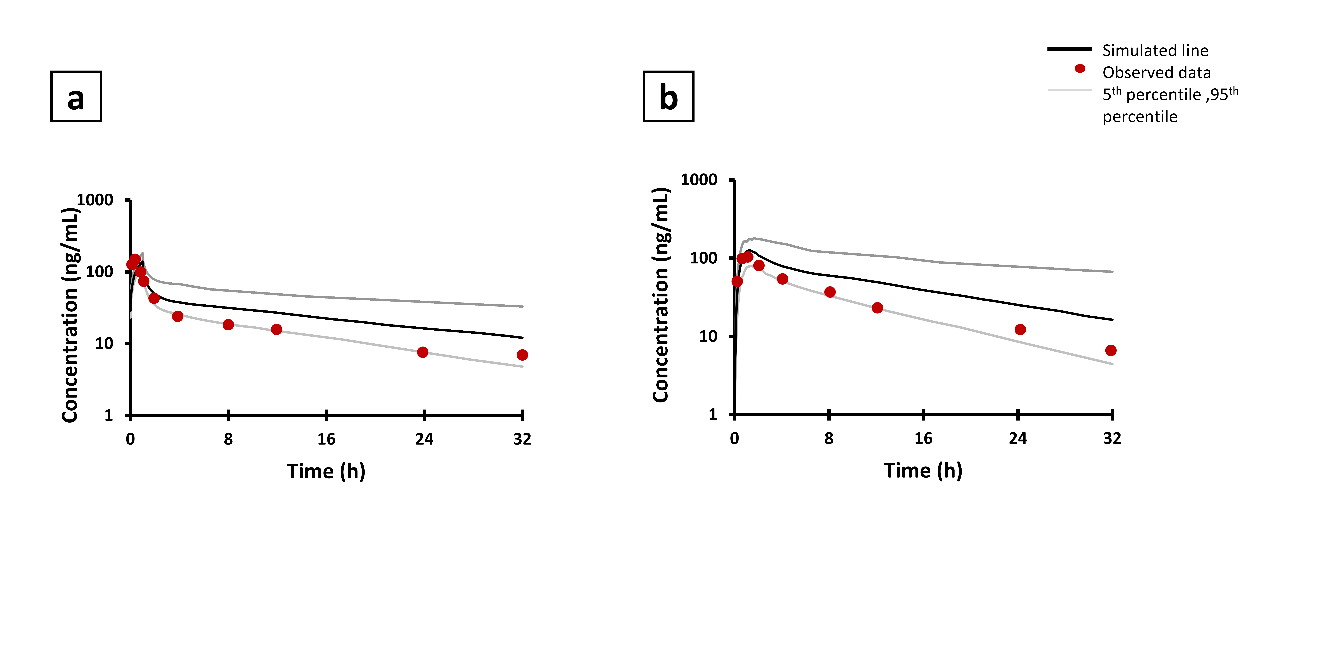
Supplementary Fig. S2** Visual inspection for carvedilol in cirrhotic patients

a) Median plasma concentration time profile of carvedilol after a single IV infusion dose of 12.5 mg over one hour (10 trials of 6 cirrhotic patients (CP-C), 40-76 years, 16.7% females) ^13^ .

b) Median plasma concentration time profile of carvedilol after a single oral dose of 25 mg (10 trials of 6 cirrhotic patients (CP-C), 40-76 years, 16.7% females) ^13^.

CP-A; Child -Pugh A, CP-B; Child -Pugh B, CP-C; Child -Pugh C


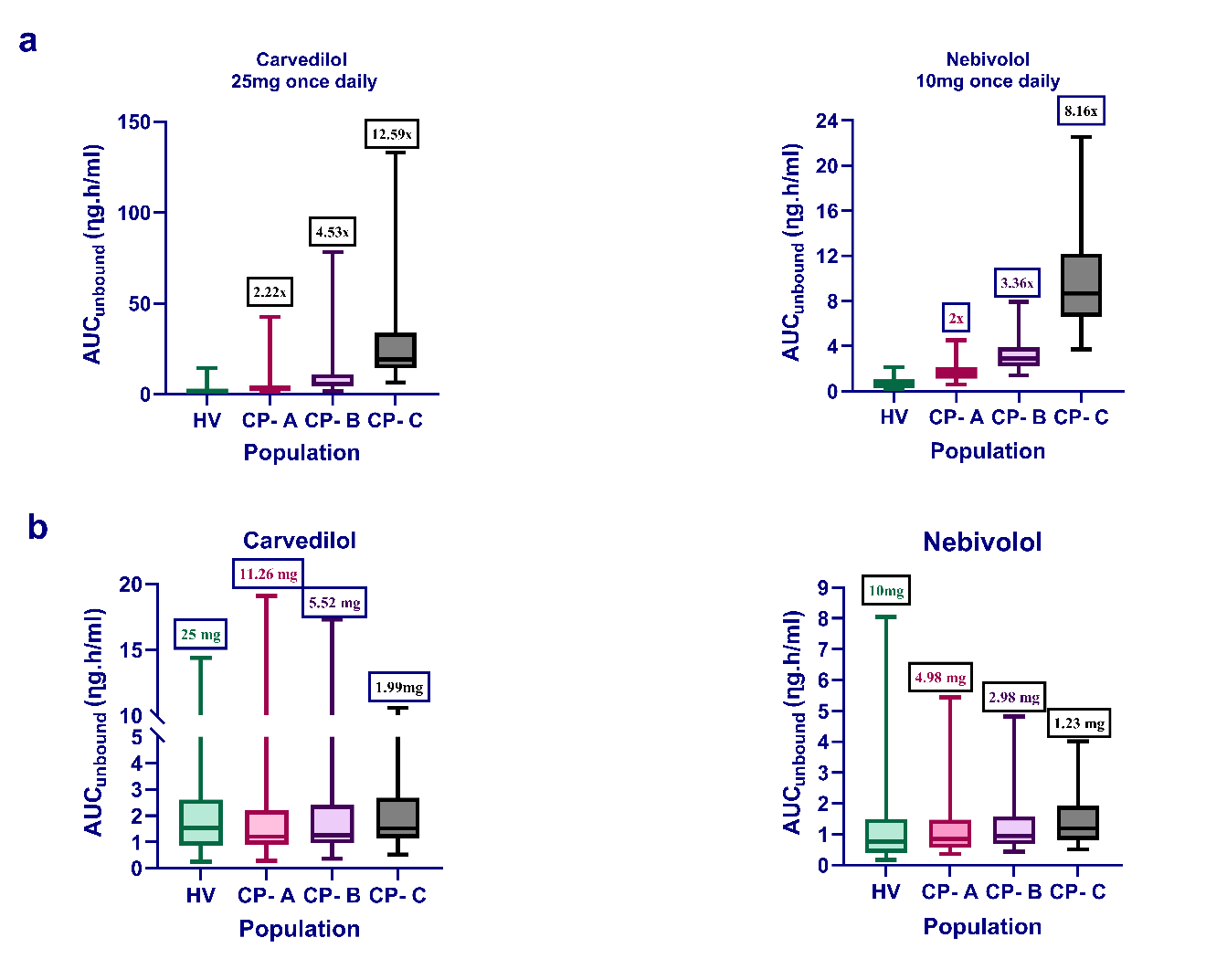


**Supplementary Fig. 3** Boxplots showing the predicted area under the curve (AUC0-last) with 5th – 95th percentiles in healthy and cirrhotic patients. Panel (a) refers to the predicted AUC _unbound_ before applying dose reduction of carvedilol (25 mg once daily) and nebivolol (10 mg once daily) to healthy cirrhosis patients. Panel (b) refers to the predicted AUC _unbound_ after a reduction in the administered doses of carvedilol and nebivolol to cirrhotic patients. HV; Healthy volunteers, CP-A; Child -Pugh A, CP-B; Child -Pugh B, CP-C; Child -Pugh C

Supplementary Table S9. Basal clinical characteristics of study patients (n = 40 analysed; 4 patients from CP-A withdrew during follow-up; see CONSORT flow diagram, Fig 1)

| Parameters | Mean ± SD  Number (percentage) |
| --- | --- |
| Age | **55.90 ± 6.27** |
| Weight | **73.83 ± 4.1** |
| Height | **1.72 ± 0.06** |
| BMI (kg/ m^2^) | **24.87 ± 1.04** |
| Gender | |
| Male (n%) | **26 (65%)** |
| Female (n%) | **14 (35%)** |
| Cause | |
| Hepatitis C (n%) | **26 (65%)** |
| NAFLD (n%) | **14 (35%)** |
| Alb (g/dl) | **3.35 ± 0.3** |
| Bilirubin (mg/dL) | **1.77 ± 0.6** |
| INR | **1.29 ± 0.14** |
| ALT (U/L) | **34.63 ± 6.73** |
| AST (U/L) | **42.16 ± 5.80** |
| Scr (mg/dL) | **0.95 ± 0.14** |
| BUN (mg/dL) | **21.75 ± 2.87** |
| FBG (mg/dL) | **106.78 ± 8.61** |
| SBP (mmHg) | **146.73 ± 6.56** |
| DBP (mmHg) | **88.1± 3.00** |
| MAP (mmHg) | **107.65 ± 3.77** |
| HR (beats/min) | **88.85± 3.22** |
| PVD (mm) | **14.2 ± 1.14** |
| PVV (cm/s) | **15.82± 1.14** |
| CI (cm²/(cm/s)) | **0.102 ± 0.023** |
| HARI | **0.743 ± 0.048** |
| MLVI | **21.48 ± 2.86** |
| Platelets count (×10³/µL) | **141.31± 7.38** |
| Hb (g/dL) | **11.72 ± 0.70** |
| WBCs (×10³/µL) | **5.19 ± 0.56** |

BMI; Body Mass Index, n%; Number and Percentage, NAFLD; Non-Alcoholic Fatty Liver Disease, Alb; Albumin INR; International Normalized Ratio, ALT; Alanine Aminotransferase, AST; Aspartate Aminotransferase, Scr; Serum Creatinine, BUN; Blood Urea Nitrogen, FBG; Fasting Blood Glucose, SBP; Systolic Blood Pressure, DBP; Diastolic Blood Pressure, MAP; Mean Arterial Pressure, HR; Heart Rate, PVD; Portal Vein Diameter, PVV; Portal Vein Velocity, CI; Congestion Index, HARI; Hepatic Artery Resistive Index, MLVI; Modified Liver Vascular Index, Hb; Hemoglobin WBCs; White Blood Cells

Supplementary Table S10: Pathophysiological changes between healthy populations and cirrhotic patients with different classes applied in Simcyp version 14

| Parameter | Gender | Population | | | | Reference |
| --- | --- | --- | --- | --- | --- | --- |
|  |  | Healthy | CP-A | CP-B | CP-C | ^19^ |
| Hepatic arterial blood flow rate (%) | Male / female | 6.27 | 7.78 | 7.88 | 8.81 |  |
| Portal blood flow rate (%) | Male | 19.1 | 15.13 | 9.32 | 7.51 |  |
|  | Female | 21.66 | 17.27 | 10.44 | 8.61 |  |
| Villous blood flow rate (%) | Male / female | 6 | 6.64 | 7 | 8.5 |  |
| Cardiac output  (L/h) | Male/ female | 303.82 | 350.69 | 399.31 | 428.82 |  |
| Liver volume (L) | Male / female | 1.5 | 1.49 | 1.2 | 1 |  |
| (Alb) (g/L) | Male / female | 44.64 | 40.36 | 33.57 | 25.35 |  |
| Hematocrit (%) | Male / female | 40.22 | 35.6 | 32.34 | 30.1 |  |
| Mean fasting gastric emptying time (h) | Male | 121.61 | 84.64 | 69.64 | 66.96 |  |
|  | | | | | |  |
| CYP1A2 (pmol/mg/protein) | | 73 | 48.67 | 36.42 | 24.4 |  |
| CYP2D6 (pmol/mg/protein) | | 61 | 43.58 | 28.06 | 6.71 |  |
| CYP2C9 (pmol/mg/protein) | | 52 | 31.34 | 12.84 | 6.1 |  |
| CYP2E1 (pmol/mg/protein) | | 8 | 4.179 | 2.09 | 0.84 |  |

CYP; Cytochrome, Supplementary Table S10 is provided for comparison with the values used in earlier work (Rasool et al. 2017, Simcyp v14); the present study uses the updated v22 values shown in Table S3.

**Supplementary Table S11 Comparison of PBPK-predicted doses and nearest commercially available doses based on unbound AUC exposure ratios**

| Child-Pugh Class | Drug (Commercial Dose) | Predicted AUC_unbound_ mean (ng/mL·hr) for commercial dose | Drug (Predicted Dose) | Predicted AUC_unbound_ mean (ng/mL·hr) for predicted dose | Commercial / predicted AUC_unbound_  ratio |
| --- | --- | --- | --- | --- | --- |
| A | Nebivolol (5 mg) | 1.42 | Nebivolol (4.98 mg) | 1.41 | 1 |
| B | Nebivolol (2.5 mg) | 1.18 | Nebivolol (2.98 mg) | 1.41 | 0.84 |
| A | Carvedilol (12.5 mg) | 2.32 | Carvedilol (11.26 mg) | 2.09 | 1.13 |
| B | Carvedilol (6.25 mg) | 2.36 | Carvedilol (5.52 mg) | 2.09 | 1.12 |

AUC; Area under the curve

Reference

1. Caron, G. *et al.* Structure-Lipophilicity Relationships of Neutral and Protonated β-Blockers, Part I, Intra- and Intermolecular Effects in Isotropic Solvent Systems. **82**, 1211–1222 (1999).

2. Fujimaki, M., Murakoshi, Y. & Hakusui, H. Assay and Disposition of Carvedilol Enantiomers in Humans and Monkeys: Evidence of Stereoselective Presystemic Metabolism. *J. Pharm. Sci.* **79**, 568–572 (1990).

3. Jamei, M. *et al.* Population-Based Mechanistic Prediction of Oral Drug Absorption. *AAPS J.* **11**, 225–237 (2009).

4. Bachmakov, I., Werner, U., Endress, B., Auge, D. & Fromm, M. F. Characterization of β-adrenoceptor antagonists as substrates and inhibitors of the drug transporter P-glycoprotein. *Fundam. Clin. Pharmacol.* **20**, 273–282 (2006).

5. Loftsson, T., Vogensen, S. B., Desbos, C. & Jansook, P. Carvedilol: Solubilization and cyclodextrin complexation: A technical note. *AAPS PharmSciTech* **9**, 425–430 (2008).

6. Berezhkovskiy, L. M. Volume of Distribution at Steady State for a Linear Pharmacokinetic System with Peripheral Elimination. *J. Pharm. Sci.* **93**, 1628–1640 (2004).

7. Takekuma, Y., Yagisawa, K. & Sugawara, M. Mutual Inhibition between Carvedilol Enantiomers during Racemate Glucuronidation Mediated by Human Liver and Intestinal Microsomes. *Biol. Pharm. Bull.* **35**, 151–163 (2012).

8. Gehr, T. W. B. *et al.* The pharmacokinetics of carvedilol and its metabolites after single and multiple dose oral administration in patients with hypertension and renal insufficiency. *Eur. J. Clin. Pharmacol.* **55**, 269–277 (1999).

9. Hanif, N. *et al.* Clinical pharmacokinetics of nebivolol: a systematic review. *Drug Metab. Rev.* **55**, 428–440 (2023).

10. Louis, W. J., McNeil, J. J., Workman, B. S., Drummer, O. H. & Conway, E. L. A pharmacokinetic study of carvedilol (BM 14.190) in elderly subjects: preliminary report. *J. Cardiovasc. Pharmacol.* **10 Suppl 11**, S89-93 (1987).

11. McPhillips, J. J., Schwemer, G. T., Scott, D. I., Zinny, M. & Patterson, D. Effects of Carvedilol on Blood Pressure in Patients with Mild to Moderate Hypertension. *Drugs* **36**, 82–91 (1988).

12. Rudorf, J. E. & Ehmer, B. Pharmacokinetic and Pharmacodynamic Interactions of Combined Acute Administration of Carvedilol and Hydrochlorothiazide in Hypertensive Volunteers. *Drugs* **36**, 113–117 (1988).

13. Neugebauer, G., Gabor, M. & Reiff, K. Pharmacokinetics and bioavailability of carvedilol in patients with liver cirrhosis. *Drugs* **36**, 148–154 (1988).

14. Stout, S. M. *et al.* The impact of paroxetine coadministration on stereospecific carvedilol pharmacokinetics. *J. Cardiovasc. Pharmacol. Ther.* **15**, 373–379 (2010).

15. Neugebauer, G., Akpan, W., von Möllendorff, E., Neubert, P. & Reiff, K. Pharmacokinetics and disposition of carvedilol in humans. *J. Cardiovasc. Pharmacol.* **10 Suppl 11**, S85–S88 (1987).

16. Martin, R. S. *et al.* Kinetic and thermodynamic assessment of binding of serotonin transporter inhibitors. *Journal of Pharmacology and Experimental Therapeutics* **327**, 991–1000 (2008).

17. Storelli, F., Desmeules, J. & Daali, Y. Physiologically‐Based Pharmacokinetic Modeling for the Prediction of CYP2D6‐Mediated Gene–Drug–Drug Interactions. *CPT Pharmacometrics Syst. Pharmacol.* **8**, 567 (2019).

18. Obach, R. S., Walsky, R. L. & Venkatakrishnan, K. Mechanism-Based Inactivation of Human Cytochrome P450 Enzymes and the Prediction of Drug-Drug Interactions. *Drug Metab Dispos* **35**, 246–255 (2007).

19. Rasool, M. F., Khalil, F. & Läer, S. Optimizing the Clinical Use of Carvedilol in Liver Cirrhosis Using a Physiologically Based Pharmacokinetic Modeling Approach. *Eur. J. Drug Metab. Pharmacokinet.* **42**, 383–396 (2017).
